# Gene expression network analysis identified CDK1 and KIF11 as possible key molecules in the development of colorectal cancer from normal tissues

**DOI:** 10.1186/s44342-025-00046-3

**Published:** 2025-06-02

**Authors:** Soo Bin Lee, Young Seon Noh, Ji-Wook Moon, Soohyun Sim, Sung Won Han, Eun Sun Kim, Ji-Yun Lee

**Affiliations:** 1 School of Industrial and Management Engineering, Korea University, Seoul, 02841 Republic of Korea; 2 BK21Plus Medical Science, Department of Anatomy, Korea University College of Medicine, Seoul, 02841 Republic of Korea; 3 Department of Biomedical Science, Korea University College of Medicine, Seoul, 02841 Republic of Korea; 4 Department of Pathology, Korea University College of Medicine, Seoul, 02841 Republic of Korea; 5 Department of Internal Medicine, Korea University College of Medicine, Seoul, 02841 Republic of Korea

**Keywords:** Colorectal cancer, Network analysis, CDK1, KIF11

## Abstract

**Background:**

Colorectal cancer (CRC) is one of the most common malignancies and the second most common cause of cancer-related mortality worldwide. Despite extensive research, the mechanism underlying CRC development remains unclear. This study aimed to understand the development and progression of CRC.

**Methods:**

Gene network analysis of tumors with their paired normal tissues was performed using the differentially expressed genes dataset for CRC from the Cancer Genome Atlas. Further investigation of the regulatory relationship between hub genes and tumor development was conducted by protein–protein interaction network, Gene Ontology enrichment, and Kyoto Encyclopedia of Genes and Genomes pathway analyses using the selected hub genes.

**Results:**

The network was more centered, and a common hub as well as a hub of hub genes were more connected to each other in the tumor than in the normal tissue, indicating changes in the network from normal to tumor. Eight downregulated and two upregulated hub genes (*CDK1* and *KIF11*) in the tumor were identified. Further, the regulatory pathway was altered, especially in cell cycle and cell division. All R implementation codes are available on the journal website as supplementary materials.

**Conclusions:**

Our findings may help understand the biological processes underlying tumor development and progression and suggest CDK1 and KIF11 as possible key molecules in the development of CRC.

**Supplementary Information:**

The online version contains supplementary material available at 10.1186/s44342-025-00046-3.

## Introduction

Colorectal cancer (CRC) is one of the most common malignancies and the second most common cause of cancer-related mortality worldwide [1]. The genetic basis of the development of CRC is well understood; however, studies on the relationship between genes and their network changes from the normal state to the tumor state are limited [2–4]. Here, we attempted to understand the development of CRC from the viewpoint of the relationship between changes in genes from the normal to the tumor state by network analysis using differentially expressed genes (DEG) set from the Cancer Genome Atlas (TCGA) database (https://cancergenome.nih.gov/). DEG analysis is a widely applied conventional method for gene expression profiling. However, it has certain limitations: it cannot detect interactions between genes and analyze the involvement of the most significant DEGs [5, 6]. To overcome these limitations, we combined the degree of centrality method with the DEG analysis [7]. The degree of centrality method is one of the simplest techniques for measuring the degree of the edges between a hub gene constituting a network and other genes directly connected to the hub using the number of adjacent hub genes. The degree of centrality method can be applied to identify important hub or connector genes in terms of the degree of the network and detect the distance at which genes are located from the center or from the genes acting as connectors or hubs in a network. In addition, protein–protein interactions, Gene Ontology (GO) enrichment, and Kyoto Encyclopedia of Genes and Genomes (KEGG) pathway analyses were conducted using the selected hub genes from each group to better understand the regulatory relationship between the hub genes and the biological events driving tumor development in CRC. The integration of multi-omics data has emerged as a powerful approach to identify novel therapeutic targets in CRC [8].

## Materials and methods

### Data collection and characterization

Fifty RNA sequencing datasets with the clinical information of CRC patients and paired normal data were obtained from the COADREAD cohorts in TCGA provided by FireBrowse (Version 1.1.40) [9]. The data were divided into two groups, normal vs. tumor, and used for analyses (accessed in October 2019) [10]. The clinical information from the collected dataset included age, sex, TNM stage, and radiation therapy status (Table 1).

**Table 1:** Clinical characteristics of the 50 patients with colorectal cancer collected from TCGA

Clinical characteristics		Colorectal cancer	
		Paired normal (50)	Tumor (50)
Age	Mean (SD)	69.58 ± 13.58	69.58 ± 13.58
	Median	73	73
Gender	Female	27 (27)	27 (27)
	Male	23 (23)	23 (23)
	NA	0	0
Radiation therapy	No	42 (42)	42 (42)
	Yes	2 (2)	2 (2)
	NA	6 (6)	6 (6)
Stage	I	8 (8)	8 (8)
	II	24 (24)	24 (24)
	III	9 (9)	9 (9)
	IV	8 (8)	8 (8)
	NA	1 (1)	1 (1)
T stage	t1	2 (2)	2 (2)
	t2	7 (7)	7 (7)
	t3	35 (35)	35 (35)
	t4	6 (6)	6 (6)
N stage	n0	34 (34)	34 (34)
	n1	8 (8)	8 (8)
	n2	8 (8)	8 (8)
M stage	m0	35 (35)	35 (35)
	m1	8 (8)	8 (8)
	mx	6 (6)	6 (6)
	NA	1 (1)	1 (1)

### DEG and network analysis

Initially, our dataset consisted of 20,553 genes. We filtered out genes with expression values of “0” in more than half of the samples, resulting in a selection of 17,649 genes. To identify significant differentially expressed genes (DEGs), we applied the Wilcoxon test and adjusted for multiple comparisons using the false discovery rate (FDR) method, as described by Benjamini and Hochberg (1995) [11]. After ranking the 17,649 genes based on their adjusted FDR levels, we established a threshold at the FDR level corresponding to the middle-ranked gene. Genes with an FDR level equal to or greater than this threshold were excluded, and we selected genes with an FDR level less than the threshold, which was set at 0.001. This process yielded a final set of 9427 genes. We considered this subset sufficient for network analysis, as it represented a substantial proportion of genes based on differential expression levels. A total of 9427 selected genes used for the DEG analysis were also used for gene network analysis to compare the normal and tumor by the network estimation method. The network estimation method finds probabilistic neighbors (the edge gene in a network) for each gene (the node within the network) using LASSO regression. To find the appropriate probabilistic neighbors through LASSO regressions, penalty parameter values should be obtained as 0.889165 according to the formula proposed by Meinshausen and Buhlmann [12]. LASSO regression was conducted using the R package glmnet [13]. And we confirmed the plot of connectivity versus the estimated penalty parameter values to find the appropriate number of connections for each gene (Supplementary Fig. 1) [14]. The hub genes of the network of the normal and tumor were obtained according to the degree of centrality. For further network analysis, the sum of the edge weights associated with each hub was calculated [15]. The weight indicates the partial correlation between the hub and edges; the edge that has a higher impact on the hub has the greater weight [12]. Degrees refer to the number of edges connected to the hub. The more powerful a number of connections, the more influential the network is for a particular group. Based on this value, we chose the hub gene whose weight and degree were larger than the average weight and average degree.

### The protein–protein interaction network, GO and KEGG pathway enrichment, and survival analysis

**Fig. 1:**
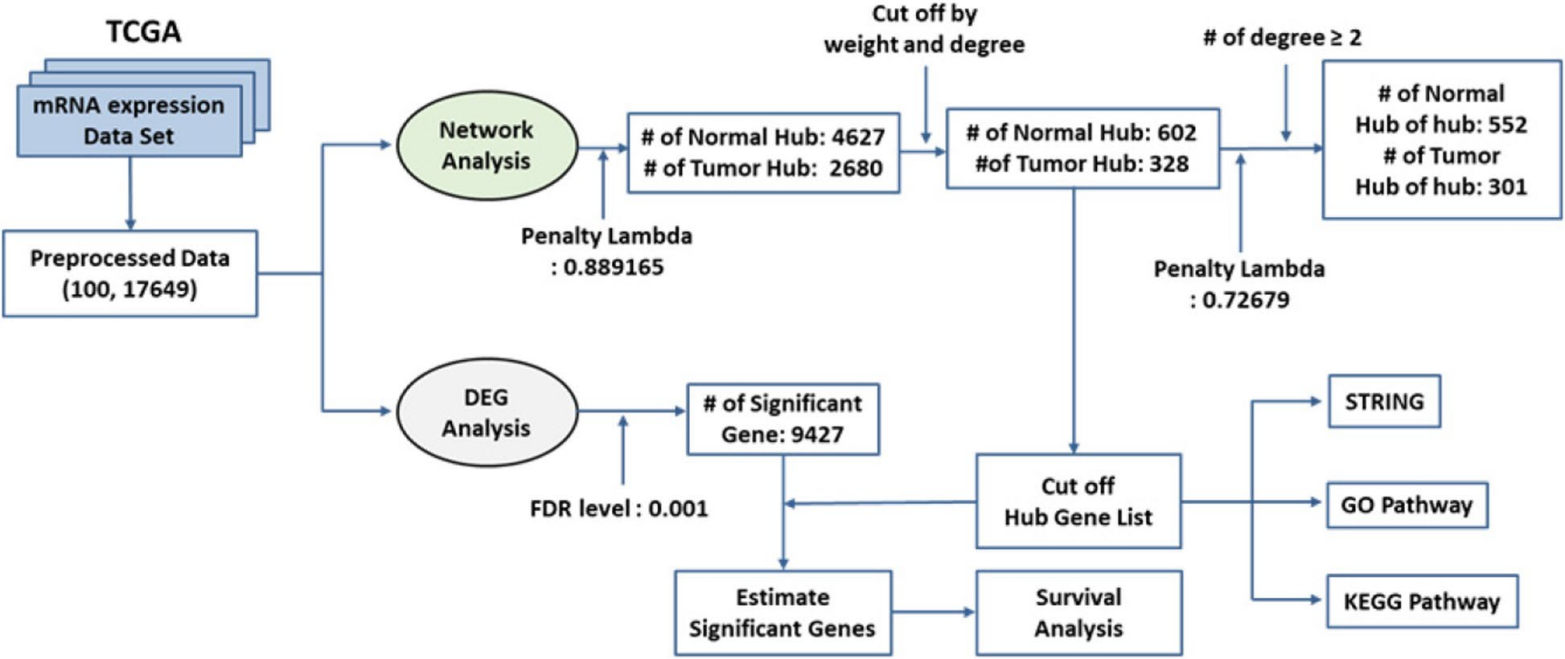
The overall process of data analysis. #Mean number

All analyses were performed as described previously [16] with minor modifications (supplementary method and Supplementary Fig. 1). The overall data process is shown in Fig. 1.

## Results

### DEG analysis

**Fig. 2:**
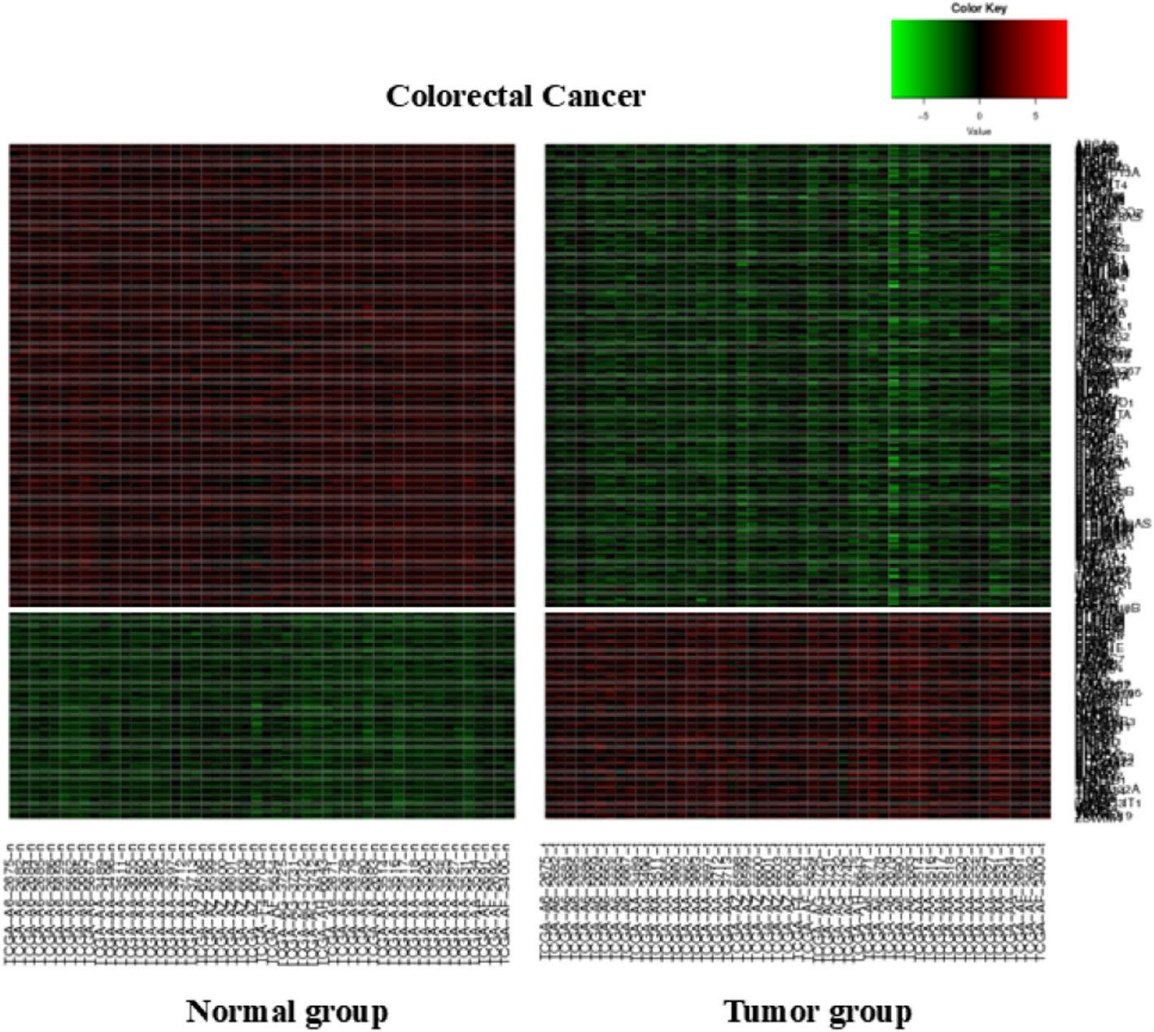
Heatmap of 244 upregulated and 112 downregulated DEGs with the lowest *p*-values, categorized into respective groups

To analyze the DEG levels between the tumor and normal tissues, a total of 9427 genes were reselected by applying FDR to control the type I error (1) [17]. An FDR level of < 0.001 was used to adjust the (adj) *p*-values in the multiple comparison test using 17,649 genes selected by the Wilcoxon test (Supplementary Table 1). The relative gene expression levels between the normal and tumor tissues were subdivided into upregulated and downregulated genes, among which the sets of upregulated (244) and downregulated (112) genes with the highest *p*-values were plotted on a heat map (Fig. 2).

### Degree of centrality analysis

**Fig. 3:**
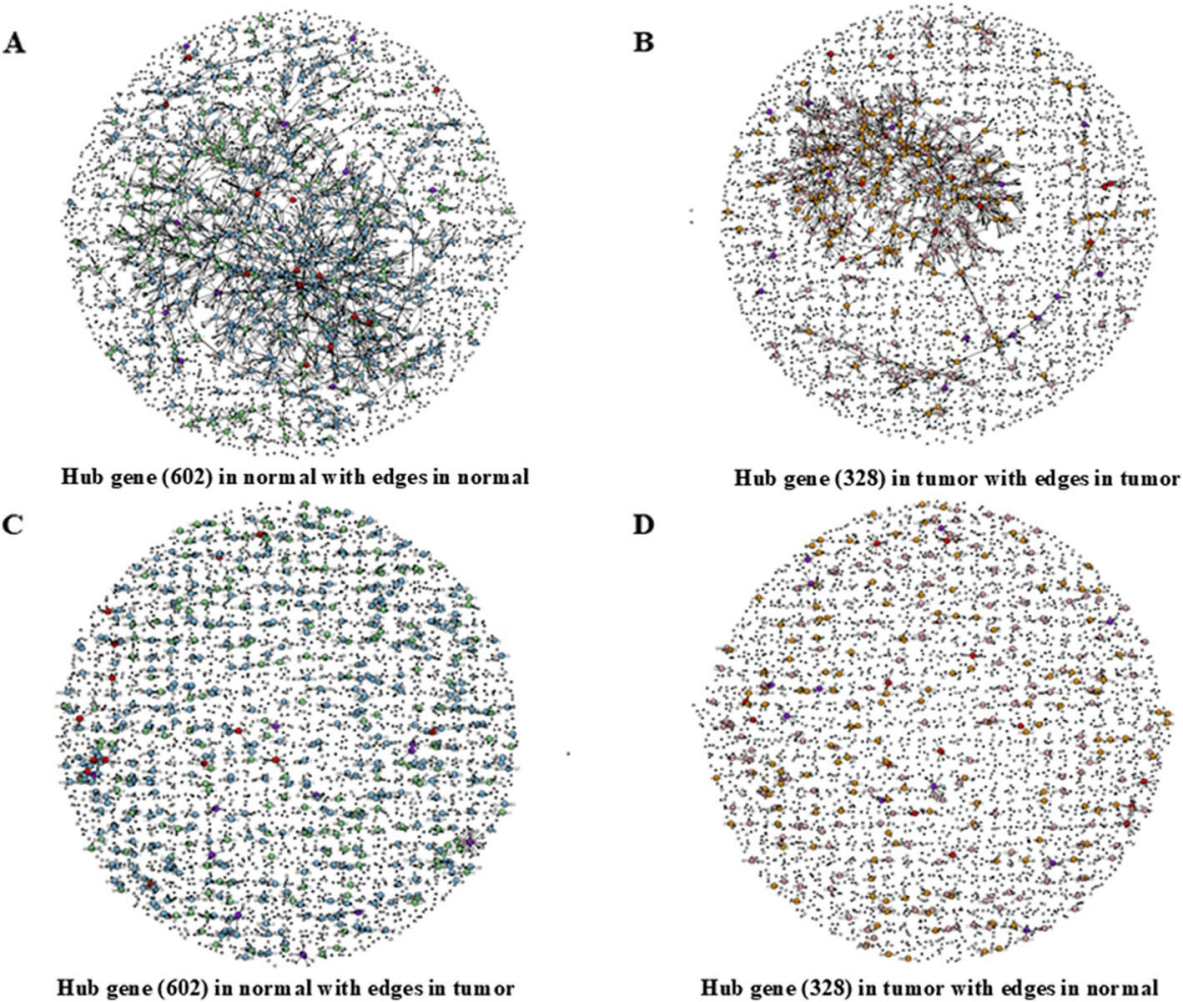
Network analysis of hub genes with their edges. **A** Hub genes (602) of the normal group applied to the gene network of the normal group. **B** Hub genes (328) of the tumor group applied to the gene network of the tumor group. **C** Hub genes (602) of the normal group applied to the gene network of the tumor group. **D** Hub genes (328) of the tumor group applied to the gene network of the normal group. The list of gene sets in the network included all hub genes and their edge genes from both the normal and tumor groups; however, the position of each gene is not the same in **A**, **B**, **C**, and **D**. Large balls indicate hub genes, and small balls indicate edge genes. Lines indicate the connection between genes. Purple indicates common hub genes that are upregulated in the tumor (downregulated in the normal tissue). Red indicates common hub genes that are downregulated in the tumor (upregulated in normal tissue). Green indicates hub genes only in the normal tissue that are downregulated in the normal tissue (upregulated in the tumor). Sky blue indicates hub genes only in the normal tissue that are upregulated in the normal tissue (downregulated in the tumor). Pink indicates hub genes only in the tumor that are upregulated in the tumor (downregulated in the normal tissue). Orange indicates hub genes only in tumor that are downregulated in the tumor (upregulated in the normal tissue). White indicates edge genes

**Fig. 4:**
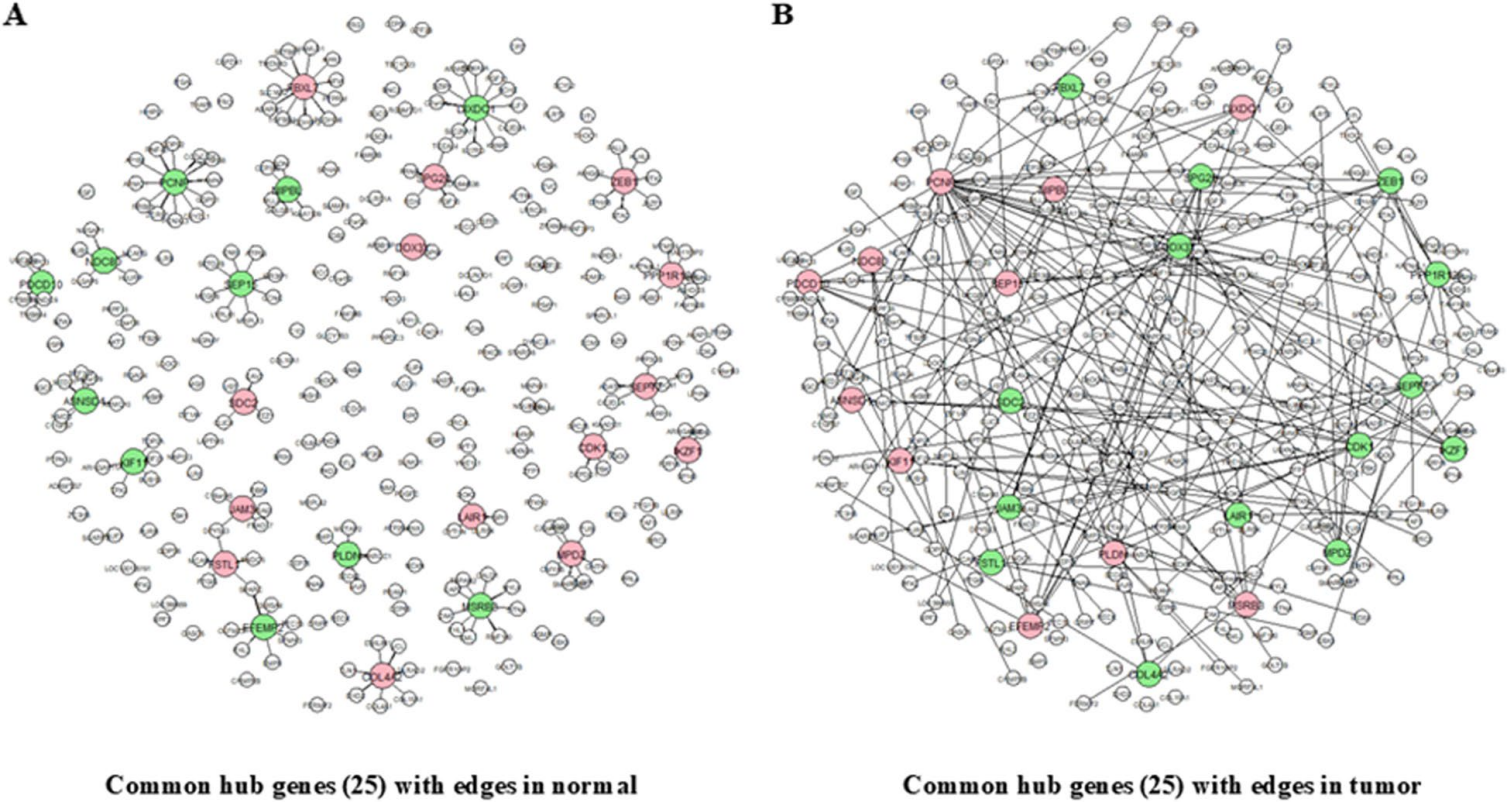
Network analysis of 25 shared hub genes showing connections (edges) specific to the normal group (**A**) and tumor group (**B**). Gene positions remain consistent between both panels for direct comparison. Large balls indicate hub genes, and small balls indicate edge genes. Lines indicate connections between genes. Pink indicates hub genes that are upregulated in the tumor (downregulated in normal tissue), and green indicates hub genes that are downregulated in the tumor (upregulated in normal tissue)

The gene expression networks of the normal and tumor tissues showed a scale-free topology (Supplementary Fig. 2), indicating that the degree of each gene was not evenly distributed and focused on a specific hub gene, thereby following the power-law distribution [18]. In the degree of centrality analysis, 4627 hub genes from normal tissue and 2680 hub genes from the tumor were calculated with at least 1 edge (neighbor) gene from the preprocessed set of 17,649 genes, which were listed and sorted in the order of degrees and weights (Supplementary Table 2A, B). The mean degree per hub gene was ~ 2.069 (range, 1–16) in the normal group and 2.018 (range, 1–29) in the tumor group. Hub genes with both weights and edges greater than or equal to the mean/average (0.0679 and 3.5 for the normal and 0.0603 and 3.4 for the tumor, respectively) were selected for further analysis. Thus, 602 hub genes (13%) were identified from the normal group, and 328 hub genes (12.2%) were identified from the tumor group (Supplementary Table 3A, B). The mean degree per hub gene was ~ 5.6 (range, 4–16) in the normal group and ~ 5.9 (range, 4–29) in the tumor group. These data indicated that the number of hub genes from the normal to the tumor state was reduced by half. In addition, the network was more gathered in the center and isolated from the peripheral network in the tumor group than the normal group (Fig. 3A, B). Such finding implies that more hub genes with their edges in the gene network play a general role in normal tissue, but the fewer hub genes, which differed from normal, with their edges in the gene network play a specific role in tumors by connecting more tightly than in normal tissue. Among the 602 hub genes in the normal group and 328 hub genes in the tumor group, 25 genes were identified as a common hub between both groups (Supplementary Table 4), representing 4.2% (25/602) in the normal group and 7.6% (25/328) in the tumor group. The mean degree for the 25 common hub genes was ~ 6.6 (range, 4–12) in the normal group and ~ 6.9 (range, 4–29) in the tumor group. Of these 25 common hub genes, 6 hub genes (24%) shared common edge genes with a range of 1–3 in both groups. In other words, 19 (76%) hub genes did not share common edge genes with a range of 4–12 in the normal and 4–29 in the tumor groups. The resulting network showed that common hub genes in the tumor were more closely linked to each other than those in the normal tissue, which were local and isolated (Fig. 4). Furthermore, the mean degree of the hub genes only in the normal group (577) was 5.6 (range, 4–16), while that only in the tumor group (303) was 5.8 (range, 4–18). The resulting network was similar to that of the original network, including 25 common hubs (Supplementary Fig. 3). These results indicated that there were differences in the gene networks between the normal and tumor states. In addition, only nine common hub genes, *PCNP*, *MSRB3*, *MPDZ*, *EFEMP2*, *ZEB1*, *NIPBL*, *SEPT7*, *SPG20*, and *CDK1*, with high degrees and whose number of edges was greater than the mean degree in both groups were selected. The number of edges of the remaining 16 common hub genes was less than the mean degree in both or one of the groups. This finding indicated that the main hub genes changed from normal to tumor state along with their edges. The representative network of each hub with their edge genes with high degrees in each group is shown and compared, showing changes in their edge genes (Supplementary Fig. 4).

A degree of centrality analysis was performed using the selected 602 hub genes of the normal group and the tumor group to investigate and compare how edge genes and degrees are altered with the same hub genes in each group (Supplementary Table 3A-1). Of the 602 hub genes in the normal group, 181 (30.1% in the normal group and 55.2% in the tumor group) were identified with a range of 1–29 edge genes in the tumor group; the edge genes in the tumor for the rest of the hub genes [421 (69.9%) in the normal group and 147 (44.8%) in the tumor group] were not identified. From the 181 hub genes with edges in both groups, the mean degree was 5.7 (range, 4–13) in the normal and 2.4 (range, 1–29) in the tumor groups. However, only 25 (13.8%) of the 181 hub genes had common edges, which denotes a mean degree of 1.1 with a range of 1–3 in both groups. Similarly, 328 hub genes in the tumor were found to be normal (Supplementary Table 3B-1). For the 328 hub genes in the tumor (38.7% in the tumor and 20.1% in the normal), edge genes were identified in the range of 1–12. For the remaining hub genes [201 (61.3%) in the tumor and 475 (78.9%) in the normal groups], edge genes could not be identified. From the 127 hub genes with edges in both groups, the mean degree was 6.0 (range, 4–29) in the tumor and 2.6 (range, 1–12) in the normal groups. However, only 15 (11.9%) of the 127 hub genes had common edges with a mean degree of 1.2 (range, 1–3) in both groups. This result implies that only 11–14% of the edge genes with the same hub gene were from each other’s groups, confirming that the gene network is very different between the normal and tumor tissue. Thus, the gene network changed from the normal to tumor state, and the network between hub genes was more dispersed when the genes were applied to the other group compared to when they were applied to their own groups (Fig. 3C, D).

### Degree of centrality analysis of only the hub genes

A network analysis using only the hub genes, without counting their edge genes, was conducted to investigate the hub of hub genes (Supplementary Table 5A, B) and to understand the relationships between hub genes, which was similar to that obtained for the hub genes. A total of 552 hubs of hub genes from the normal and 301 hub of hub genes from the tumor groups were calculated with more than 2 edge genes as a gene with 1 edge cannot be considered as a hub. The mean degree of the hub of hub genes was 4.0 (range, 2–15) in the normal and 4.2 (range, 2–18) in the tumor groups. The network analysis showed that the hub of hub genes with their edges in the tumor was more centered than that in the normal (Supplementary Fig. 5A and B). The 22 common hub genes, except *DDX3Y*, *SEP15*, and *EFEMP2*, from both groups were also common in the hub of hub genes, with the mean degree of the common hub of hub genes being 4.8 (range, 2–10) in the normal and 4.27 (range, 2–12) in the tumor. These common hub of hub genes shared almost no edge genes, with a mean degree of 1 in both groups. Network analysis showed that the common hubs of hub genes were more connected to each other in the tumor than the normal state, and the hub of hub genes was connected to their edges (Supplementary Fig. 6). The mean degree of 530 (96%) hub of hub genes only in the normal group was 3.94 (range, 2–15), while that of 279 (92.7%) hub of hub genes only in the tumor group was 4.21 (range, 2–18). As shown in the network of hub genes and only hub genes in the normal or tumor group with their edges, the network of hub of hub genes only in the normal group with their edges based on only hub genes showed a pattern similar to the network of hub genes in only normal and only tumor tissue (Supplementary Fig. 7). Furthermore, four common hub of hub genes, *PCNP*, *MPDZ*, *PPP1R12A*, and *SEPT7*, with a high degree were selected, and their numbers of edges were greater than the mean degree in each group. Of these four genes, *PCNP*, *MPDZ*, and *SEPT7* were also common hub genes with a high degree, indicating that they are important genes in the gene network, even if the edge genes had changed (Supplementary Table 5). The representative network of each hub of hub genes and the edge genes with a high degree from each group is shown in Supplementary Fig. 8.

The network of 22 common hub of hub genes was examined with edges using hub genes as well as their original edges (Supplementary Table 3) of hub genes in the normal and tumor group to determine how the network changed when the same genes were applied to the 2 groups (Supplementary Fig. 9A and B). The results were similar to those obtained with the network of hub genes with their edges in each group (Fig. 3A and B). In addition, the hub of hub genes from one group was applied to the other group. Data showed that the network of the hub of hub genes from the normal changed, and a very small number of networks existed when the same hub of hub genes was applied to the tumor (Supplementary Fig. 9C). The opposite analysis showed a consistent result, indicating that the network changed from normal to tumor state (Supplementary Fig. 9D). Taken together, even if the hub genes were common, the network changed from a normal to tumor state and became considerably different.

### Comparison of DEG sets and hub genes from each group

**Fig. 5:**
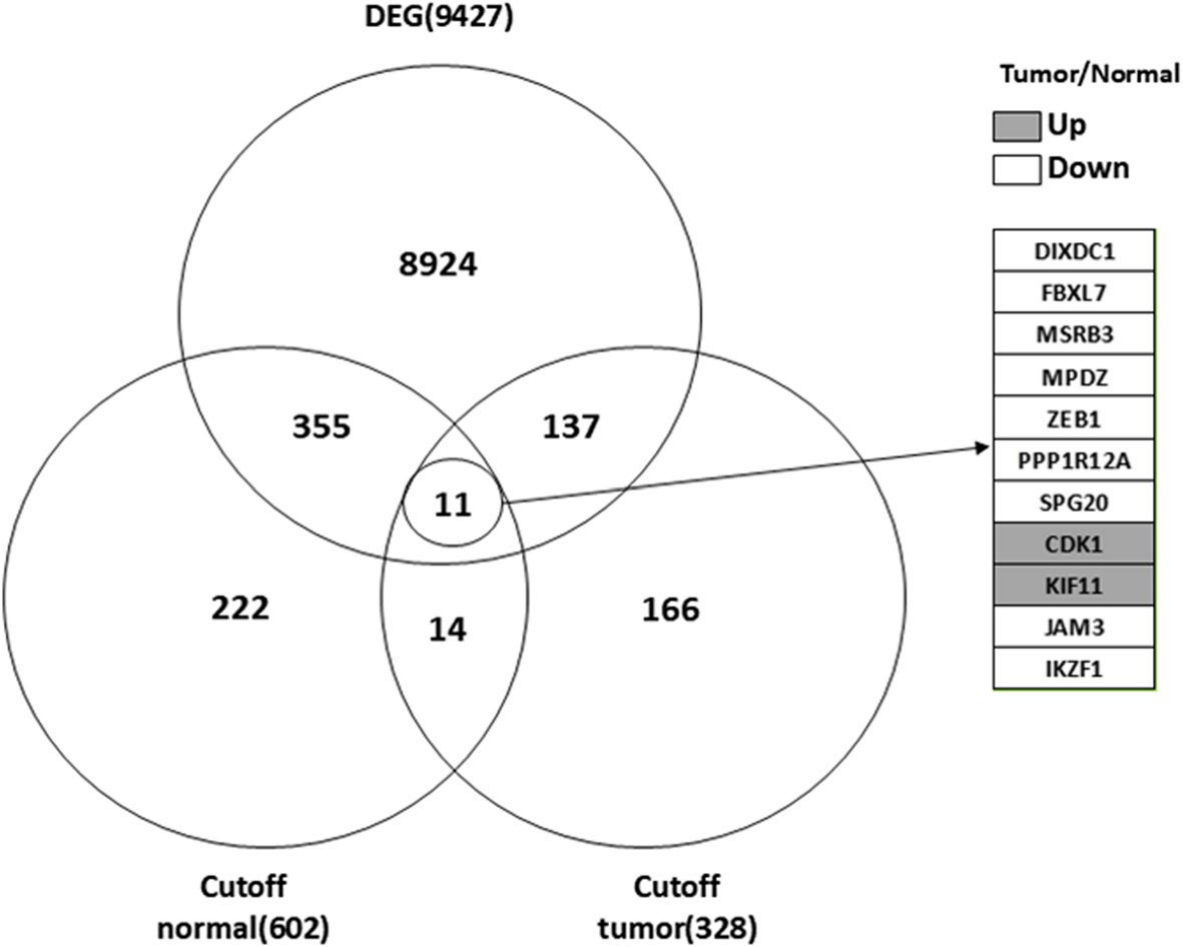
Venn diagram of genes shared across the 9427 DEG (*p* ≤ 0.001 and *p* < padj) sets and hub genes from the normal (602 ≥ mean weight: 0.0679 and ≥ mean degree: 3.5) and the tumor (328 ≥ mean weight: 0.0603 and ≥ mean degree: 3.4) groups. The number indicates the number of genes, which are listed in Table 2

Hub genes from the normal and tumor tissues were compared with the DEG set (9427 genes) to select hub genes that were highly differentially expressed between the two groups and may serve as markers to distinguish tumor from normal tissues, thereby showing potential application as prognostic markers (Supplementary Table 6). Among the 25 hub genes in both the normal and tumor tissues, 9 genes (*DIXDC1*, *FBXL7*, *MSRB3*, *MPDZ*, *ZEB1*, *PPP1R12A*, *SPG20*, *JAM3*, and *IKZF1*) were downregulated, and 2 genes (*CDK1* and *KIF11*) were upregulated in the tumor compared to the normal tissue (Fig. 5 and Table 2), whose functions are described in Supplementary Table 7. Of the 577 hub genes in the normal group, 355 genes were shared with the DEG set, 287 were downregulated, and 68 were upregulated; of the 303 genes only in the tumor, 137 genes were shared with the DEG sets, 85 were downregulated, and 52 were upregulated in the normal compared with the tumor group. Furthermore, the differences in the expression levels between the hub genes in the normal and tumor tissues were examined, and 503 hub genes were found to show significantly (*p* ≤ 0.001 and *p* ≤ padj) different expression levels between the normal and tumor group, even if these genes were not included in the 9427 DEG set (Supplementary Table 6).

**Table 2:** Selected hub genes based on a comparison of the DEG sets and hub genes from each group

	Gene	Analysis	Normal	Tumor	Relative gene expression level (tumor/normal)	*p*-value
DEG and normal and tumor	*DIXDC1*	Degree	12	5	Down	0.0000
		Expression^a^	9.194	8.026		
	*FBXL7*	Degree	11	5	Down	0.0000
		Expression^a^	7.617	6.522		
	*MSRB3*	Degree	10	8	Down	0.0000
		Expression^a^	10.070	8.123		
	*MPDZ*	Degree	7	8	Down	0.0000
		Expression^a^	7.778	6.491		
	*ZEB1*	Degree	7	9	Down	0.0000
		Expression^a^	9.343	8.127		
	*PPP1R12A*	Degree	7	5	Down	0.0000
		Expression^a^	10.352	10.106		
	*SPG20*	Degree	6	7	Down	0.0000
		Expression^a^	8.706	7.114		
	*CDK1*	Degree	6	8	Up	0.0000
		Expression^a^	8.862	10.278		
	*KIF11*	Degree	5	6	Up	0.0002
		Expression^a^	8.800	9.666		
	*JAM3*	Degree	5	4	Down	0.0000
		Expression^a^	9.283	8.038		
	*IKZF1*	Degree	4	6	Down	0.0000
		Expression^a^	8.763	7.11		

^a^Indicates log_2_ median

A survival analysis using Kaplan–Meier estimation was performed with the selected genes (supplementary methods, Supplementary Fig. 10, and Supplementary Table 8); however, none had statistical significance for the tumor group.

### Protein–protein interaction network, GO, and KEGG pathway enrichment analysis with the selected hub genes

**Fig. 6:**
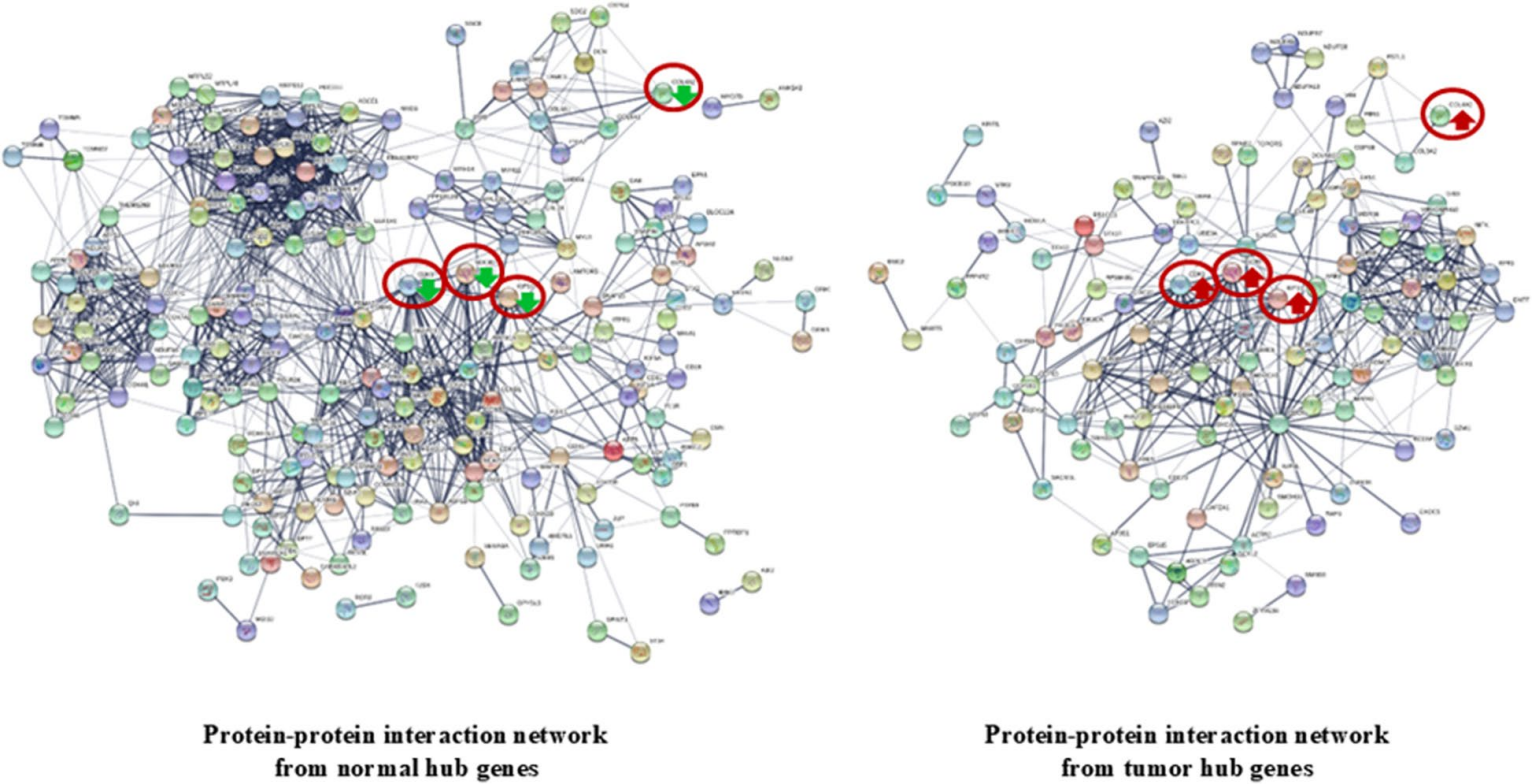
Protein–protein interaction networks among the hub genes from the normal (602) and tumor (328) groups with a confidence score 0.95, as analyzed by STRING. Balls represent proteins, and lines represent interactions between proteins. A red circle around a ball indicates genes shared by both groups. Red and green arrows indicate upregulation and downregulation, respectively

STRING analysis was performed to further explore the physical and functional protein interaction networks among the hub genes from each group. A protein–protein interaction network of 193 hub genes (32.06%) in the normal and 105 hub genes (32.01%) in the tumor group was found, with a confidence score > 0.95. Of these hub genes, only four genes, *CDK1*, *NDC80*, *KIF11*, and *COL4A2*, were shared between the two groups, whereas 189 hub genes from the normal group replaced 101 different hub genes from the tumor group, resulting in protein–protein network differences between the normal and tumor groups (Fig. 6 and Supplementary Table 9). However, interactions between the shared hub genes did not differ between the groups and were retained from the normal to the tumor group. In addition, 113 hub genes from the normal group, 48 hub genes from the tumor group, and 2 hub genes (CDK1 and KIF11) from both groups were included in the 9427 DEG (*p* ≤ 0.001 and *p* ≤ padj) sets, indicating differential expression between the normal and tumor tissues (Supplementary Table 9).

**Fig. 7:**
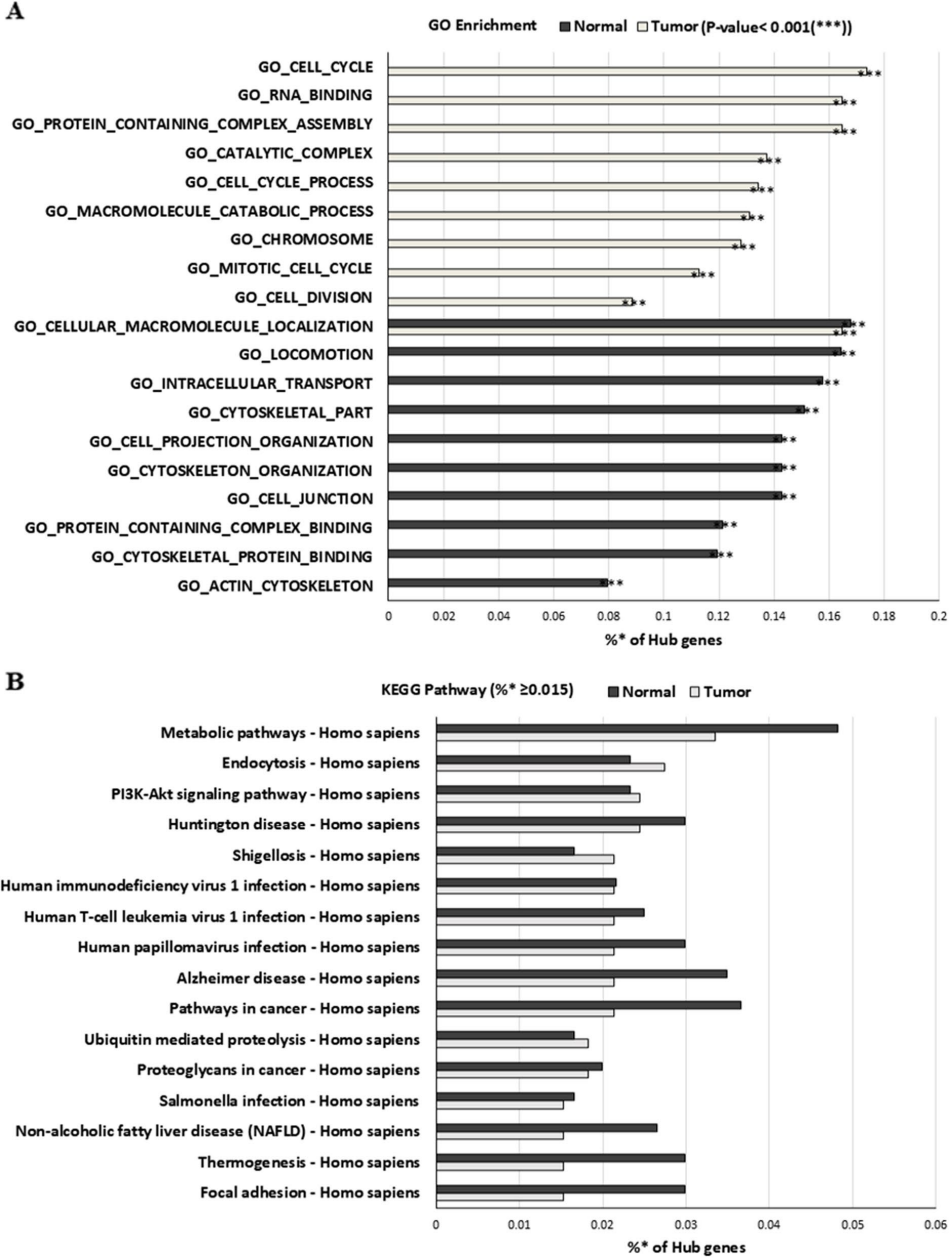
Top 10 enriched GO terms (**A**) and KEGG pathway (**B**) with more than 0.015% of the hub genes involved (searched using 602 hub genes from the normal group and 328 hub genes from the tumor group). *Indicates the proportion of the number of genes: [number of hub genes involved in this pathway/number of total hub genes from the normal and tumor group] × 100

To examine the characteristics of the hub genes from each group, functional classification of the hub genes was performed using the GO tool. The top 100 most significantly enriched GO terms for biological processes in each group were determined (Supplementary Table 10). Of these 100 enriched GO terms, 30 were common among both groups, and 70 differed between the groups. The top 10 most highly enriched GO terms from each group were selected and compared (Fig. 7A). Only one GO term, cellular macromolecule localization, was shared between both groups, and the remaining nine enriched GO terms belonged to each group, namely cell cycle- and cell division-related GO terms in the tumor group and cytoskeleton-related GO terms in the normal group. Such finding indicates that the biological function was altered between normal and tumor tissues.

KEGG pathway analysis was also performed to further understand the biological functions of these genes. A total of 242 pathways from the normal group and 191 pathways from the tumor group were enriched (Supplementary Table 11). Of these pathways, 181 pathways were common between both groups, 61 pathways (24.2%) were enriched only in the normal group, and 10 pathways (3.97%) were enriched only in the tumor group. Pathways where more than 0.015% of the hub genes were involved were compared between the normal and tumor tissues (Fig. 7B), highlighting the differences at the level of the metabolic, cancer, and focal adhesion pathways.

## Discussion

Network analysis using DEG sets of CRC showed network changes from the normal to tumor state, which was more centered, and a common hub as well as a hub of hub genes were more connected to each other in the tumor than normal state. The hub genes with a high degree in the normal group did not consist of hub genes with a high degree in the tumor group, with only < 10% of a hub or hub of hub genes being shared between groups, indicating changes in the hub genes from the normal to tumor state. Among the shared common hub genes, one interesting gene was *PCNP*, which was identified as a hub as well as a hub of hub genes with a high degree in both groups. PCNP is known to be associated with cell cycle control and may be involved in tumorigenesis [19, 20]; however, no reports have suggested its role in colon cancer. Moreover, PCNP was selected as the hub as well as a hub of hub genes in the lymph node ( +) and lymph node ( −) group network analysis in our previous study [16]. These results indicate that PCNP maintained its role as an important hub gene, and changes in its edge genes may have induced functional changes depending on the state of cells, which needs to be further investigated.

We identified nine downregulated and two upregulated hub genes in the tumor that may play important roles in the development and progression of normal cells to cancer cells. Of these 11 genes, only *MPDZ* was a common hub as well as a hub of hub genes with a high degree in both groups and was downregulated in the tumor. MPDZ encodes a protein with multiple PDZ domains, which are hallmarks of protein–protein interactions. However, its role in tumor development has not been studied, except in one study that suggested that endothelial-specific inactivation of MPDZ leads to an excessively branched and poorly functional vessel network, resulting in tumor hypoxia and enhanced tumor angiogenesis. The study also suggested that MPDZ can act as a novel modulator of Notch signaling by controlling ligand recruitment to adherent junctions [21]. The expression level of the downregulated genes was not consistent with that reported previously; this is because the roles of these genes differ based on the state and/or environment of cells and type of tumor, which requires further investigation. However, the upregulation of two significantly upregulated common hub genes, *CDK1*, which is a well-known cyclin-dependent kinase1 and key regulator of the cell cycle, and *KIF11*, which encodes a motor protein that belongs to the kinesin-like protein family and an important plyer in cell mitosis, has been consistently reported to be associated with cancer cell progression and poor prognosis of several malignant tumors, including CRC [22–24]. Based on a survival analysis, their expression levels were not significantly associated with the survival rate in this study; however, this could be due to the small number of samples included. Additionally, the regulatory relationships among the hub genes with respect to biological processes were analyzed by STRING, GO, and KEGG, which supported the finding from network analysis that the most significant changes from normal to tumor state occurred in the cell cycle and cell mitosis processes. Because we only used gene expression data for network construction, further research is required to confirm the roles of these genes in CRC development. Nonetheless, the results of this network analysis may help improve our understanding of the biological events underlying CRC development from normal tissues. Emerging recent evidence suggests that targeting CDK1 and KIF11 could be a promising therapeutic strategy for CRC and other cancers [25–28]. For instance, research on other cancers has shown that targeting specific pathways, like HER2 in solid tumors, can be effective in treatment [29]. Additionally, the role of metabolic enzymes in cancer, such as the moonlighting function of enolase-1 in promoting choline phospholipid metabolism and tumor proliferation, underscores the complexity of cancer biology [30]. Currently, a CDK1 inhibitor is under development, and certain compounds, such as RO3306, have demonstrated potential in preclinical models by targeting CDK1-related pathways [31, 32]. Additionally, agents targeting KIF11, such as SRI36666, have been identified and may serve as potential therapeutic options for inhibiting tumor progression in CRC [33].

To achieve a comprehensive understanding of CRC pathogenesis, it is crucial to consider the interplay between environmental factors, lifestyle choices, and genetic predispositions. However, TCGA data does not fully encapsulate these factors, potentially introducing biases that constitute a limitation of our study. Therefore, further analysis integrating these factors, coupled with validation through additional methods such as in vivo xenograft experiments and in vitro mechanistic studies, may provide more profound insights into CRC development and facilitate the identification of potential therapeutic targets or preventive strategies.

## Supplementary Information

Supplementary Material 1: Supplementary method: 1.1 Survival analysis. Supplementary Figure 1. Number of connections against lambda in the normal (A) and tumor (B) groups. Red dots indicate a lambda value of 0.889165, which was selected in our network analysis for appropriate number of connections for each gene. Supplementary Figure 2. Linear relationship between log degree and the log number of nodes. A. Normal (R^2^ = 0.92). B. Tumor (R^2^ = 0.93). The network in the normal and tumor groups shows a scale free topology. Supplementary Figure 3. Network analysis of hub genes only in the normal group and only in the tumor group with their edges. A. Hub genes (577) only in the normal group applied to the normal group. B. Hub genes (303) only in the tumor group applied to the tumor group. The list of gene set in the network includes hub genes except common hub genes of the normal and tumor groups, and their edges from both the normal and tumor groups (but the position of each gene is not the same between A and B). Large balls indicate hub genes, and small balls indicate edge genes. Lines indicate connection between genes. Pink indicates hub genes that are upregulated in the tumor group (down regulated in the normal group), and green indicates hub genes that are downregulated in the tumor group (upregulated in the normal group). White indicates edge genes. Supplementary Figure 4. Representative six hub genes with their edge genes calculated using the degree centrality analysis of the normal and tumor groups. A. Two common hub genes in both groups (A), only in the normal group (B), and only in the tumor group (C). Green fill indicates downregulated genes in the DEG analysis, red fill indicates upregulated genes in the DEG analysis, and red font indicates common genes in both groups. Edge width: coefficient power. Supplementary Figure 5. Network analysis of hub of hub genes with their edges using only hub genes in each group. A. Hub of hub genes (552) of the normal applied to the normal, B. Hub of hub genes (301) of the tumor applied to the tumor. The list of gene set in the network included all hub genes and their edges from both the normal and tumor, but the position of each gene was not the same between A and B. Large balls indicate the hubs of hub genes, and small balls indicate edge genes (=hub gene which are not the hub of hub). Lines indicate the connection between genes. Purple indicates common hubs of hub genes that are upregulated in the tumor (downregulated in the normal). Red indicates common hubs of hub genes that are downregulated in the tumor (downregulated in the normal). Green indicates the hubs of hub genes only in the normal that are downregulated in the normal (upregulated in the tumor). Sky blue indicates the hubs of hub genes only in the normal that are upregulated in the normal (downregulated in the tumor). Pink indicates the hub of hub genes only in the tumor that are upregulated in the tumor (downregulated in the normal). Orange indicates the hubs of hub genes only in the tumor group that are downregulated in the tumor (upregulated in the normal). White indicates edge genes. Supplementary Figure 6. Network analysis of the 22 common hubs of hub genes with their edges in the normal and tumor using only the hub genes. The list of gene set in the network included 22 common hubs of hub genes and their edges from both the normal and tumor, and the position of each gene is the same in A and B. Large balls indicate the hubs of hub genes, and small balls indicate edge genes. Pink indicates the hubs of hub genes that are upregulated in the tumor (downregulated in the normal), and green indicates the hubs of hub genes that are downregulated in the tumor (upregulated in the normal). Supplementary Figure 7. Network analysis of the hub of hub genes only in the normal group and only in the tumor group with their edges. A. Hub of hub genes (530) only in the normal group applied in the normal group, B. Hub of hub genes (279) only in the tumor group applied in the tumor group. The list of gene set in the network includes the hubs of hub genes except common hubs of hub genes of the normal and tumor groups and their edges only from the hub genes from both the normal and tumor groups, but the position of each gene is not the same between A and B. Large balls indicate the hub of hub genes, and small balls indicate the edge genes using only hub genes. Lines indicate the connection/link between genes. Pink indicates the hubs of hub genes that are upregulated in the tumor group (downregulated in the normal group), and green indicates the hubs of hub genes that are downregulated in the tumor group (upregulated in the normal group). White indicates edge genes. Supplementary Figure 8. Representative six hubs of hub genes with its edge genes from only hub genes calculated using the degree centrality analysis of the normal and tumor groups. Two hub of hub genes in both groups (A), only in the normal group (B), and only in the tumor group (C). Green fill indicates downregulated genes in the DEG analysis, red fill indicates upregulated genes in the DEG analysis, and red font indicates common genes in both groups. Edge width: coefficient power. Supplementary Figure 9. Network analysis of the hub of hub genes with edges from the hub genes and edges from the original edges of the hub genes in each group. Hub of hub genes (552) of the normal group applied into the normal group including hub genes as well as original edges (Supplementary table 3A) of hub genes. B. Hub of hub genes (301) in the tumor group applied into the tumor group including hub genes as well as original edges (Supplementary table 3B) from hub genes. C. Hub of hub genes (552) in the normal group applied to the tumor group including hub genes as well as original edges (Supplementary table 3B) of hub genes. D. Hub of hub genes (301) in the tumor group applied to the normal group including hub genes as well as original edges (Supplementary table 3A) of hub genes. The list of gene set in the network included all hub genes and their edges from both the normal and tumor groups, but the position of each gene is not same in A, B, C, and D. The largest ball indicates the common hub of hub genes, the medium-sized ball indicates hub genes, and the smallest ball indicates edges genes of hub genes. Lines indicate the connection between genes. Purple indicates common hubs of hub genes that are upregulated in the tumor group (downregulated in the normal group). Red indicates the common hubs of hub genes that are downregulated in the tumor group (downregulated in the normal group). Green indicates hub genes in the normal group that are downregulated in the normal group (upregulated in the tumor group). Sky blue indicates hub genes in the normal group that are upregulated in the normal group (downregulated in the tumor group). Pink indicates hub genes in the tumor group that are upregulated in the tumor group (downregulated in the normal group). Orange indicates hub genes in the tumor group that are downregulated in the tumor group (upregulated in the normal group). White indicates edge genes. Supplementary Figure 10. Kaplan–Meier survival curve of the selected hub genes. A. Survival curve for each gene, B. Boxplot with DEGs for each gene.

Supplementary Material 2: Supplementary Table 1. 9,427 genes selected (*p*-value < 0.001, *p*-value < adj_*p*-value) from DEG analysis of the tumor and their paired normal group with colorectal cancer collected from TCGA. Bold genes indicate the gene with the highest p-value drawn in the heat map. * indicates adj_*p*-value = i/m×FDR Level, (i: rank, m: the number of total variable, FDR Level: 0.001). Supplementary Table 2A. Degree of centrality analysis of the hub genes (4,627) with their edges from the normal group. Supplementary Table 2B. Degree of centrality analysis of the hub genes (2,680) with their edges from the tumor group. Supplementary Table 3A. Degree centrality analysis of the selected hub (602) genes with their edges in the normal group after cutting off based on the mean weight (≥0.0679) and average degree (≥ 3.5) for further analysis. Green filling indicates 25 common hub genes between the normal (Supplementary Table 3A) and tumor (Supplementary Table 3B) groups. Orange filling indicates common edges in common hub between the normal (Supplementary Table 3A) and tumor (Supplementary Table 3B) groups. Hub genes in bold (181) indicate common the hub genes between Supplementary Table 3A and 3A-1. Blue genes in bold indicate common edges of common hub genes between Supplementary Table 3A and 3A-1. Supplementary Table 3A-1. Degree of centrality analysis of the 602 hub genes for the normal group (Supplementary Table 3A) in the tumor group (Supplementary Table 2B). Hub genes in bold (181) indicate common hub genes between Supplementary Table 3A and 3A-1. Blue genes in bold indicate common edges of common hub genes between Supplementary Table 3A and 3A-1. Supplementary Table 3B. Degree centrality analysis of the selected hub (328) genes with their edges in the tumor group after cutting off based on weight (≥0.0603) and average degree (≥ 3.4) for further analysis. Green filling indicates 25 common hub genes between the normal group (Supplementary Table 3B) and the tumor group (Supplementary Table 3A). Orange filling indicates common edges in the common hub between the normal (Supplementary Table 3B) and tumor (Supplementary Table 3A) groups. Hub genes in bold (127) indicate common hub genes between Supplementary Table 3B and 3B-1. Blue genes in bold indicate common edges of common hub genes between Supplementary Table 3B and 3B-1. Supplementary Table 3B-1. Degree of centrality analysis of the 328 hub genes for the tumor group (Supplementary Table 3B) in the normal group (Supplementary Table 2A). Hub genes in bold (127) indicate common hub genes between Supplementary Table 3B and 3B-1. Bolded blue genes indicate common edges of common hub genes between Supplementary Table 3B and 3B-1. Supplementary Table 4. Summary of common and different hub genes in the tumor and paired normal groups. Supplementary Table 5A. Degree of centrality analysis of the selected 552 hub of hub genes with their edge genes from only the 602 hub genes by discounting their edge genes in the normal group. Green filling indicates 22 common hub genes between the normal (Supplementary Table 5A) and tumor (Supplementary Table 5B) groups. Orange filling indicates common edges in the common hub between the normal (Supplementary Table 5A) and tumor (Supplementary Table 5B) groups. Supplementary Table 5B. Degree of centrality analysis of the selected 301 hub of hub genes with their edge genes from only the 321 hub genes by discounting their edge genes in the tumor group. Green filling indicates 22 common hub genes between the normal (Supplementary Table 5A) and tumor (Supplementary Table 5B) groups. Orange filling indicates common edges in the common hub between the normal (Supplementary Table 5A) and tumor (Supplementary Table 5B) groups. Supplementary Table 6. DEG of hub genes in the normal and tumor groups. Yellow filling indicates 503 significant genes obtained using the FDR level (+: *p* ≤ 0.001 & p ≤ p_adj_). Red indicates 25 common hub genes in the normal and tumor groups. 0 indicates no hub in each group. * indicates adj p-value. adj p-value=i/m×FDR Level, (i: rank, m: the number of total variable, FDR Level: 0.001). Supplementary Table 7. Description and references of selected hub genes by comparison DEG sets and hub genes from each group. Supplementary Table 8. Survival analysis of the selected hub genes. Supplementary Table 9. List of hub genes investigated (602 of hub genes from normal and 328 of hub genes from tumor) by STRING analysis (Confidence score ≥ 0.95). Yellow filling indicates DEG significance (*p* ≤ 0.001 & *p* ≤ p_adj_). Supplementary Table 10. Enriched GO terms obtained upon searching 602 hub genes from the normal and 328 hub genes from the tumor groups. * [Number of hub genes involved in this pathway/number of total hub genes from the normal or tumor groups] × 100. Supplementary Table 11. Enriched pathways based on KEGG pathway analysis of the 602 hub genes from the normal and 328 hub genes from the tumor. * [Number of hub genes involved in this pathway/number of total hub genes from normal or tumor] × 100. * [Number of hub genes involved in this pathway/number of total hub genes from normal or tumor] × 100.

## Data Availability

No datasets were generated or analysed during the current study.
