# Supplementary material for: Gene expression network analysis identified CDK1 and KIF11 as possible key molecules in the development of colorectal cancer from normal tissues
Source: Genomics Inform. 2025 Jun 2;23:15. doi: 10.1186/s44342-025-00046-3 (PMC12128336; doi:10.1186/s44342-025-00046-3)
Supplement: Supplementary file 1 — Supplementary Material 1: Supplementary method: 1.1 Survival analysis. Supplementary Figure 1. Number of connections against lambda in the normal (A) and tumor (B) groups. Red dots indicate a lambda value of 0.889165, which was selected in our network analysis for appropriate number of connections for each gene. Supplementary Figure 2. Linear relationship between log degree and the log number of nodes. A. Normal (R2 = 0.92). B. Tumor (R2 = 0.93). The network in the normal and tumor groups shows a scale free topology. Supplementary Figure 3. Network analysis of hub genes only in the normal group and only in the tumor group with their edges. A. Hub genes (577) only in the normal group applied to the normal group. B. Hub genes (303) only in the tumor group applied to the tumor group. The list of gene set in the network includes hub genes except common hub genes of the normal and tumor groups, and their edges from both the normal and tumor groups (but the position of each gene is not the same between A and B). Large balls indicate hub genes, and small balls indicate edge genes. Lines indicate connection between genes. Pink indicates hub genes that are upregulated in the tumor group (down regulated in the normal group), and green indicates hub genes that are downregulated in the tumor group (upregulated in the normal group). White indicates edge genes. Supplementary Figure 4. Representative six hub genes with their edge genes calculated using the degree centrality analysis of the normal and tumor groups. A. Two common hub genes in both groups (A), only in the normal group (B), and only in the tumor group (C). Green fill indicates downregulated genes in the DEG analysis, red fill indicates upregulated genes in the DEG analysis, and red font indicates common genes in both groups. Edge width: coefficient power. Supplementary Figure 5. Network analysis of hub of hub genes with their edges using only hub genes in each group. A. Hub of hub genes (552) of the normal applied to [file 44342_2025_46_MOESM1_ESM.docx]

**Gene expression network analysis identified CDK1 and KIF11 as possible key molecules in the development of colorectal cancer from normal tissues**

Soo Bin Lee^1^, Young Seon Noh^1^, Ji-Wook Moon^2^, Soohyun Sim^3,4^, Sung Won Han^1^, Eun Sun Kim^5^, Ji-Yun Lee^4^

^1^School of Industrial and Management Engineering, Korea University, Seoul 02841, Republic of Korea

^2^BK21Plus Medical Science, Department of Anatomy, Korea University College of Medicine, Seoul 02841, Republic of Korea

^3^Department of Biomedical Science, Korea University College of Medicine, Seoul 02841, Republic of Korea

^4^Department of Pathology, Korea University College of Medicine, Seoul 02841, Republic of Korea

^5^Department of Internal Medicine, Korea University College of Medicine, Seoul 02841, Republic of Korea

**Contents**

1. **Supplementary Methods •••••••••••••••••••••••••••••••••••••••••••••••••••••••••••••••••••••••••••• 1**
   1. **Survival analysis**
2. **Supplementary Figures 1 – 8 •••••••••••••••••••••••••••••••••••••••••••••••••••••••••••••••••••••••• 2**
3. **Supplementary Table list 1 – 11 ••••••••••••••••••••••••••••••••••••••••••••••••••••••••••••••••••••• 17**

**1. Supplementary Methods**

.**1.1 Survival analysis**

Kaplan–Meier plots were used to estimate the survival rates (Goel et al., 2010). A multivariate analysis was used to evaluate whether the groups clustered by the expression levels of selected genes were an independent prognostic factor for overall survival. A p-value of less than 0.05 was considered statistically significant.

**2. Supplementary Figures**


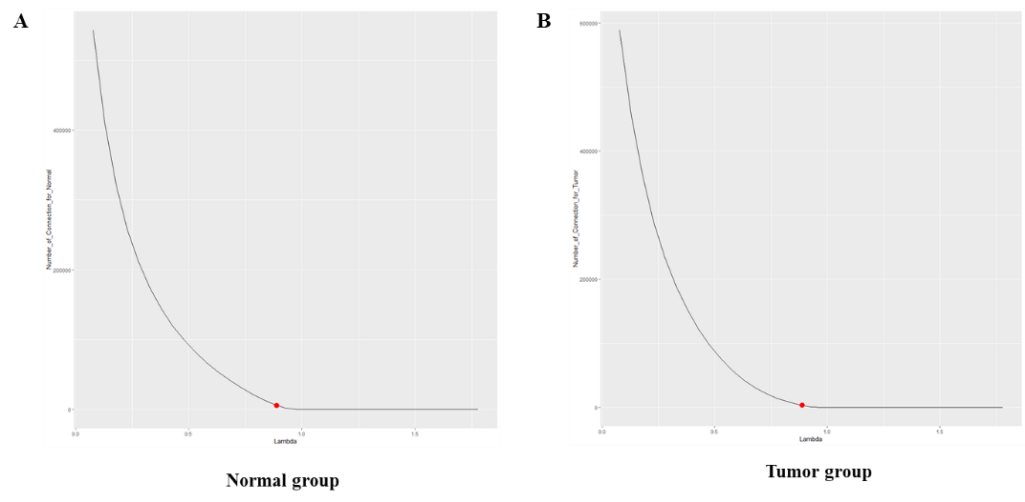


**Supplementary Figure 1. Number of connections against lambda in the normal (A) and tumor (B) groups.** Red dots indicate a lambda value of 0.889165, which was selected in our network analysis for appropriate number of connections for each gene.


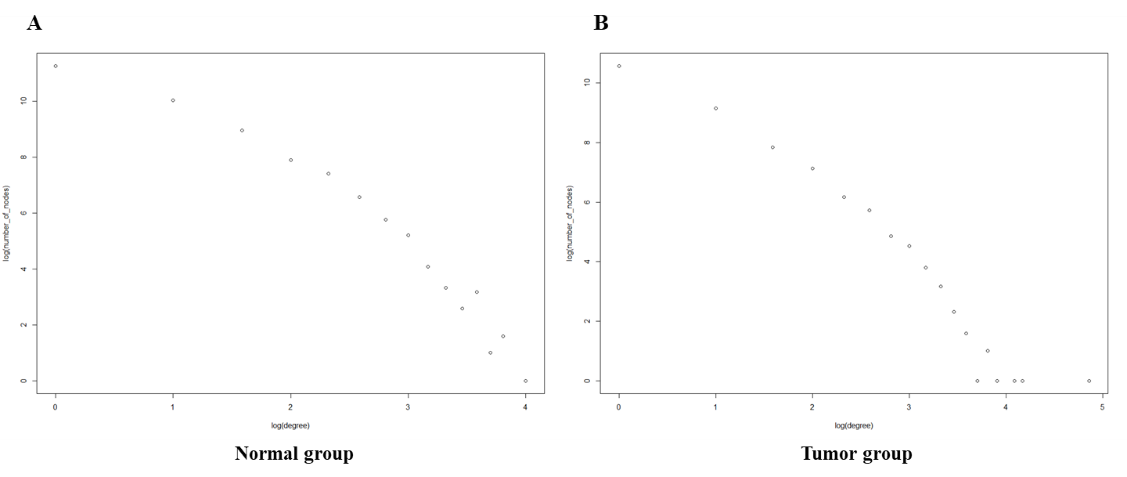


**Supplementary Figure 2. Linear relationship between log degree and the log number of nodes.** A. Normal (R^2^ = 0.92). B. Tumor (R^2^ = 0.93). The network in the normal and tumor groups shows a scale free topology.


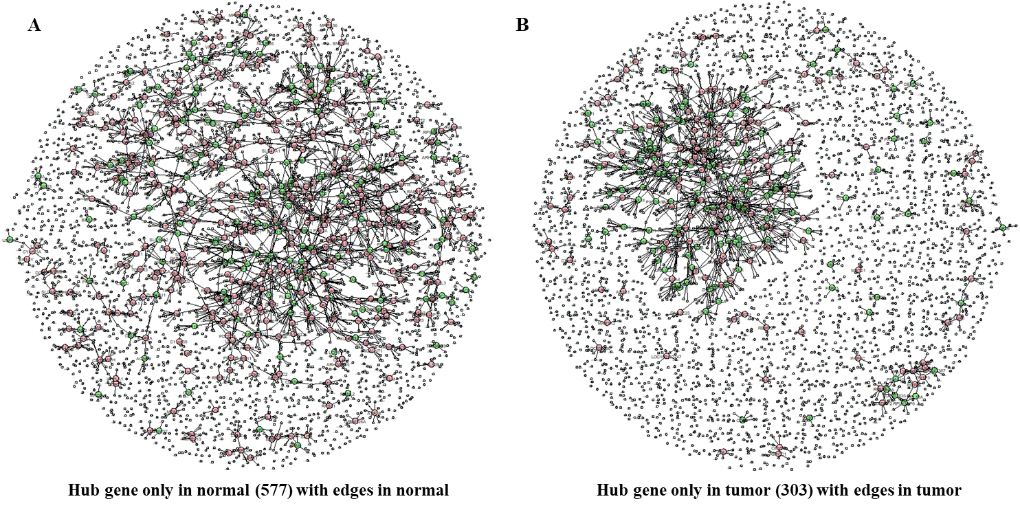


**Supplementary Figure 3. Network analysis of hub genes only in the normal group and only in the tumor group with their edges**. A. Hub genes (577) only in the normal group applied to the normal group. B. Hub genes (303) only in the tumor group applied to the tumor group. The list of gene set in the network includes hub genes except common hub genes of the normal and tumor groups, and their edges from both the normal and tumor groups (but the position of each gene is not the same between A and B). Large balls indicate hub genes, and small balls indicate edge genes. Lines indicate connection between genes. Pink indicates hub genes that are upregulated in the tumor group (down regulated in the normal group), and green indicates hub genes that are downregulated in the tumor group (upregulated in the normal group). White indicates edge genes.

Supplementary Figure 4.


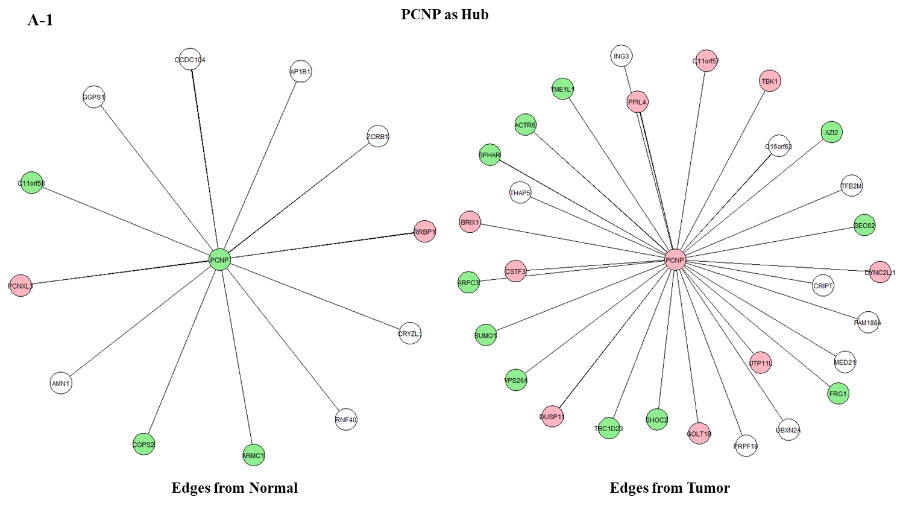


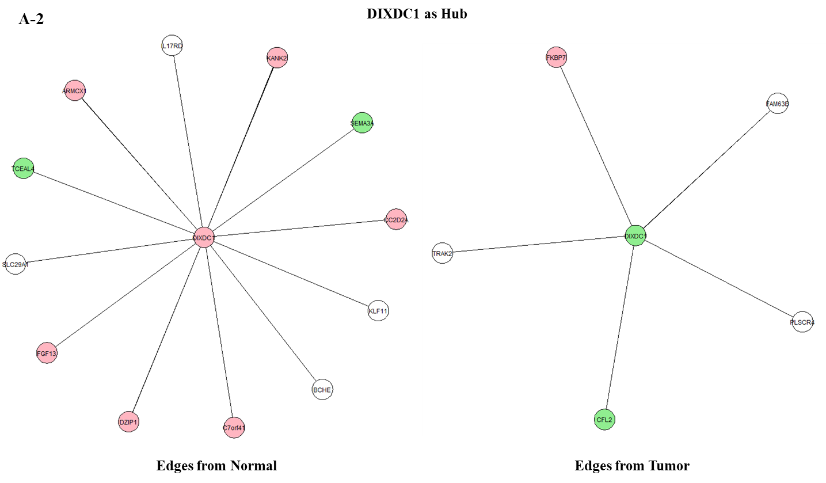


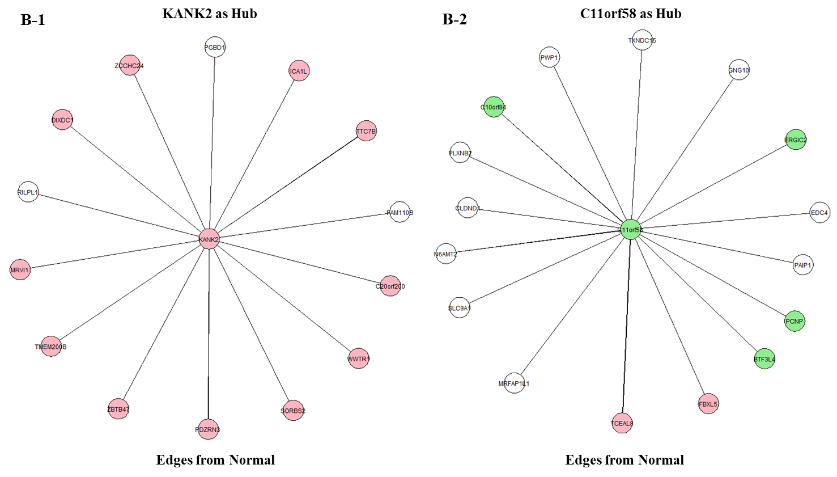


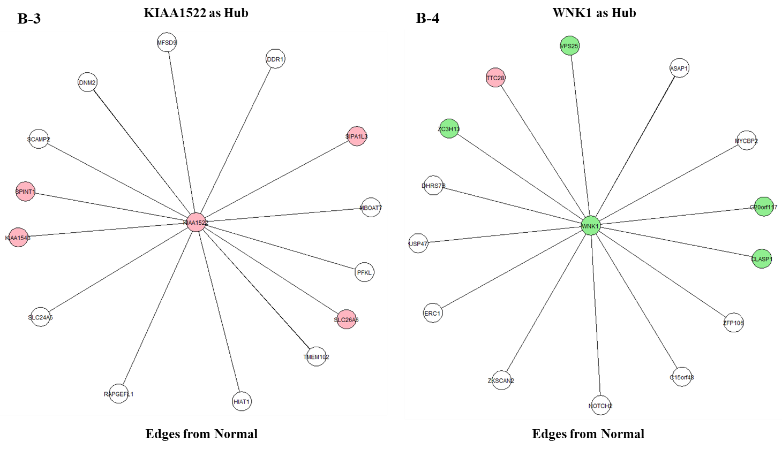


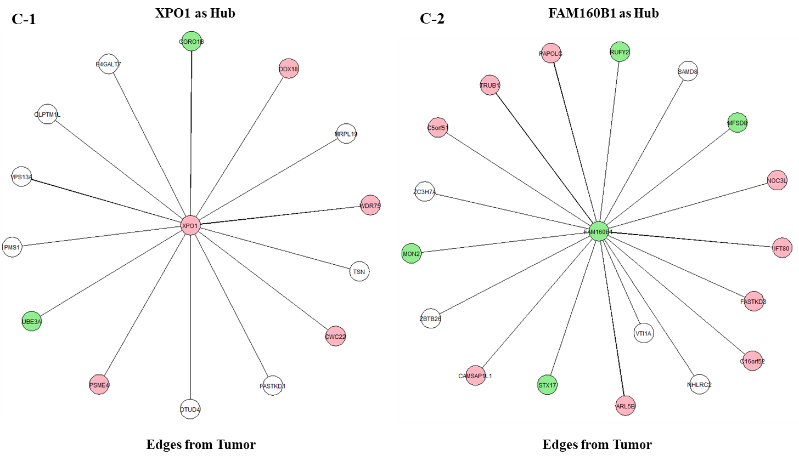


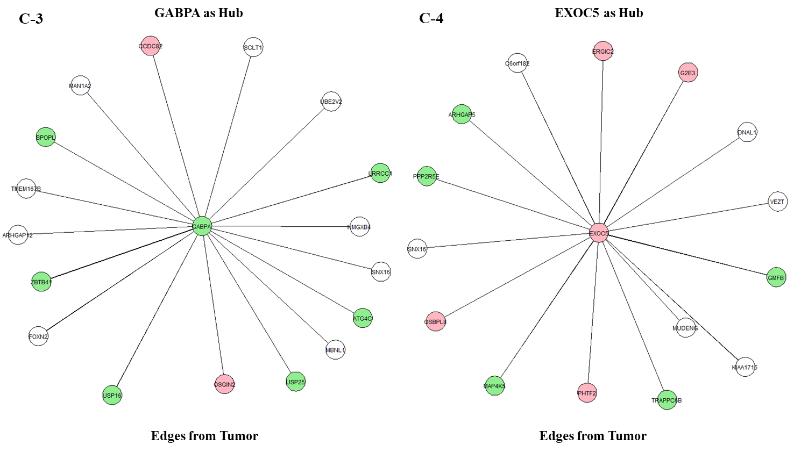


**Supplementary Figure 4. Representative six hub genes with their edge genes calculated using the degree centrality analysis of the normal and tumor groups.** A. Two common hub genes in both groups (A), only in the normal group (B), and only in the tumor group (C). Green fill indicates downregulated genes in the DEG analysis, red fill indicates upregulated genes in the DEG analysis, and red font indicates common genes in both groups. Edge width: coefficient power.


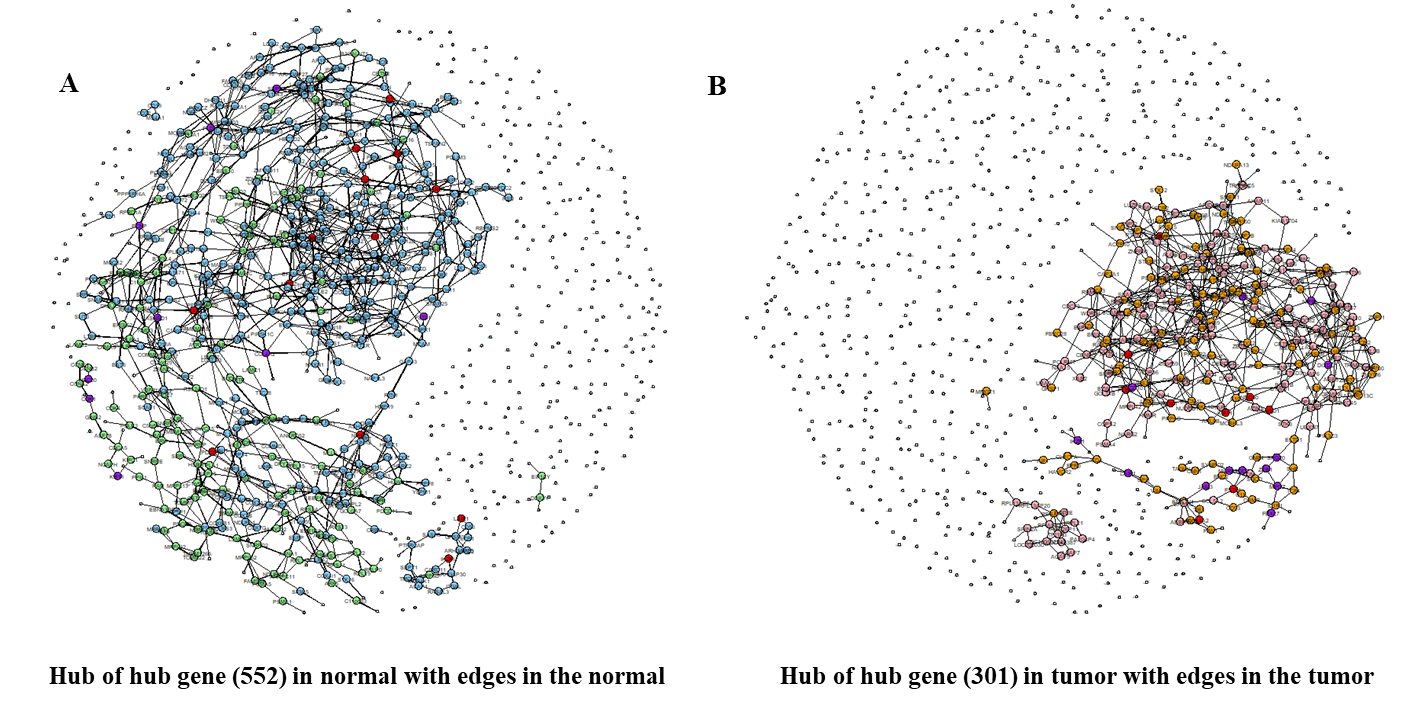


**Supplementary Figure 5. Network analysis of hub of hub genes with their edges using only hub genes in each group.** A. Hub of hub genes (552) of the normal applied to the normal, B. Hub of hub genes (301) of the tumor applied to the tumor. The list of gene set in the network included all hub genes and their edges from both the normal and tumor, but the position of each gene was not the same between A and B. Large balls indicate the hubs of hub genes, and small balls indicate edge genes (=hub gene which are not the hub of hub). Lines indicate the connection between genes. Purple indicates common hubs of hub genes that are upregulated in the tumor (downregulated in the normal). Red indicates common hubs of hub genes that are downregulated in the tumor (downregulated in the normal). Green indicates the hubs of hub genes only in the normal that are downregulated in the normal (upregulated in the tumor). Sky blue indicates the hubs of hub genes only in the normal that are upregulated in the normal (downregulated in the tumor). Pink indicates the hub of hub genes only in the tumor that are upregulated in the tumor (downregulated in the normal). Orange indicates the hubs of hub genes only in the tumor group that are downregulated in the tumor (upregulated in the normal). White indicates edge genes


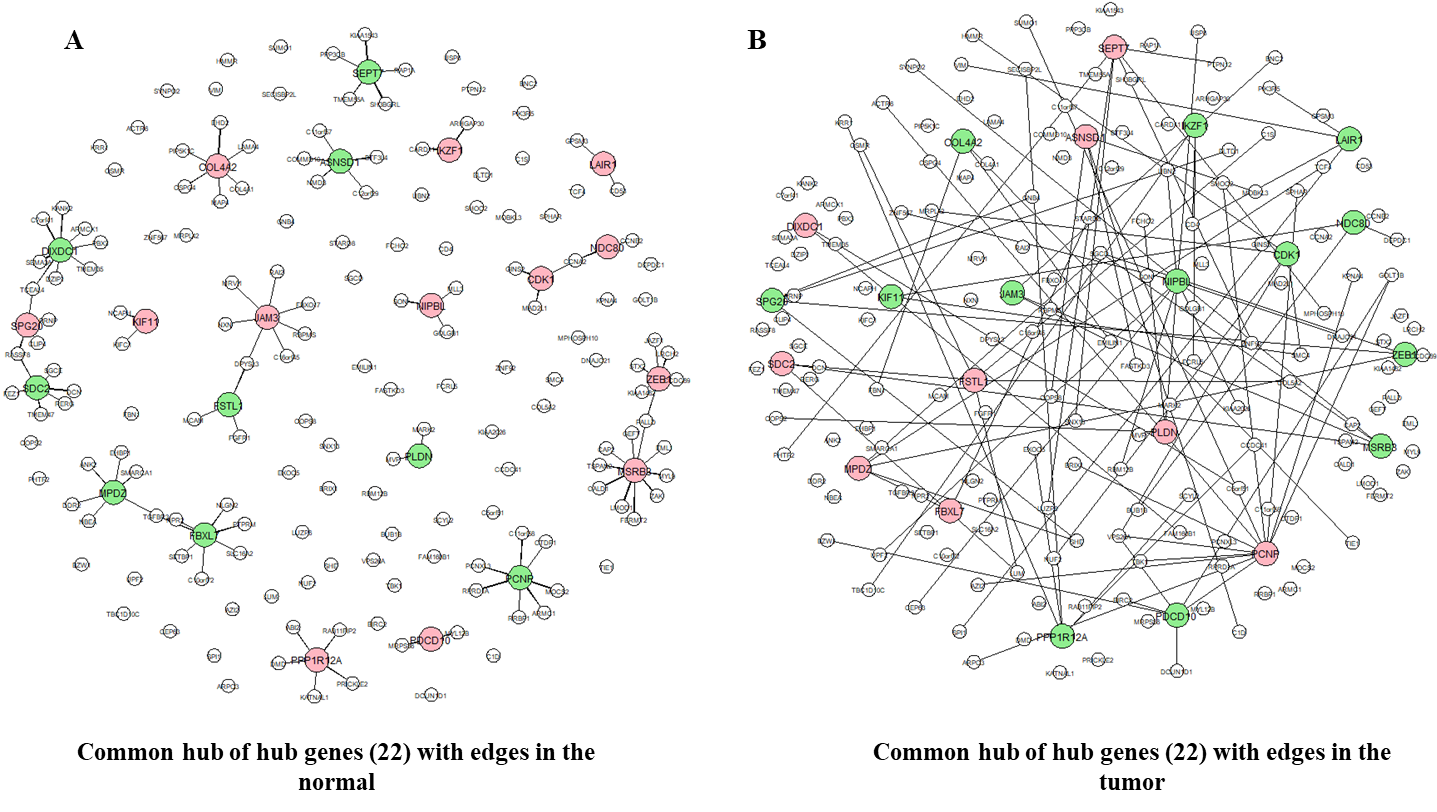


**Supplementary Figure 6. Network analysis of the 22 common hubs of hub genes with their edges in the normal and tumor using only the hub genes.** The list of gene set in the network included 22 common hubs of hub genes and their edges from both the normal and tumor, and the position of each gene is the same in A and B. Large balls indicate the hubs of hub genes, and small balls indicate edge genes. Pink indicates the hubs of hub genes that are upregulated in the tumor (downregulated in the normal), and green indicates the hubs of hub genes that are downregulated in the tumor (upregulated in the normal).


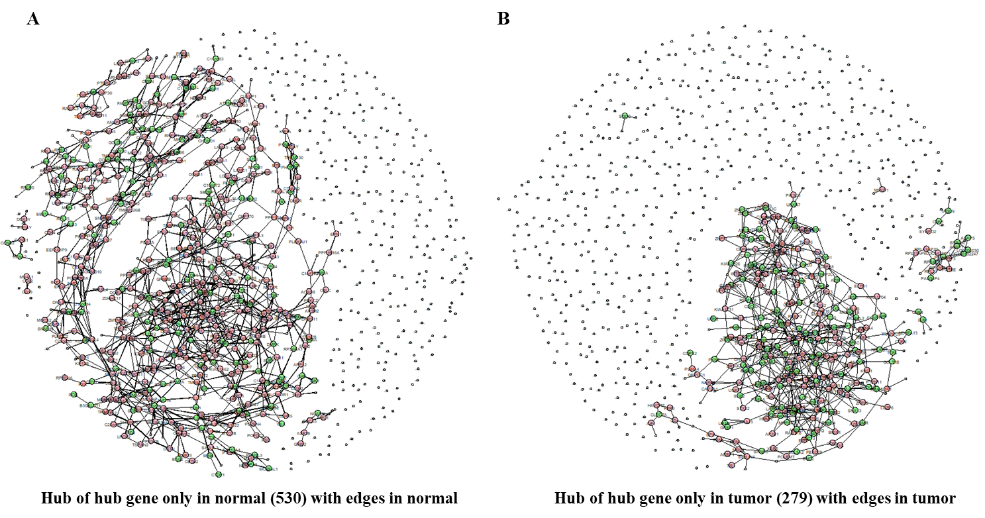


**Supplementary Figure 7. Network analysis of the hub of hub genes only in the normal group and only in the tumor group with their edges**. A. Hub of hub genes (530) only in the normal group applied in the normal group, B. Hub of hub genes (279) only in the tumor group applied in the tumor group. The list of gene set in the network includes the hubs of hub genes except common hubs of hub genes of the normal and tumor groups and their edges only from the hub genes from both the normal and tumor groups, but the position of each gene is not the same between A and B. Large balls indicate the hub of hub genes, and small balls indicate the edge genes using only hub genes. Lines indicate the connection/link between genes. Pink indicates the hubs of hub genes that are upregulated in the tumor group (downregulated in the normal group), and green indicates the hubs of hub genes that are downregulated in the tumor group (upregulated in the normal group). White indicates edge genes.

Supplementary Figure 8


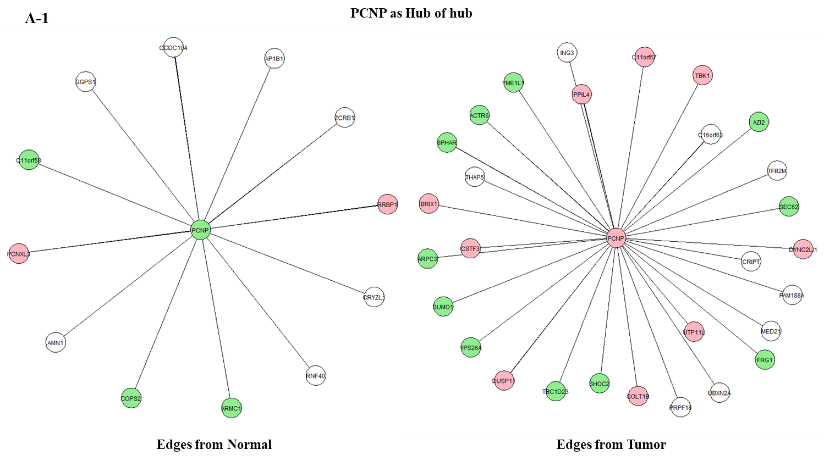


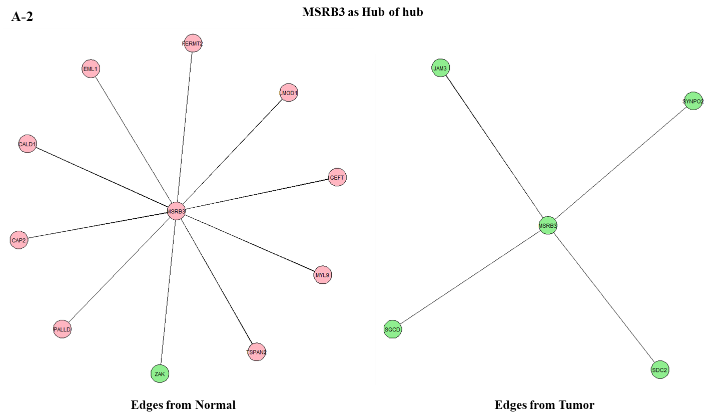


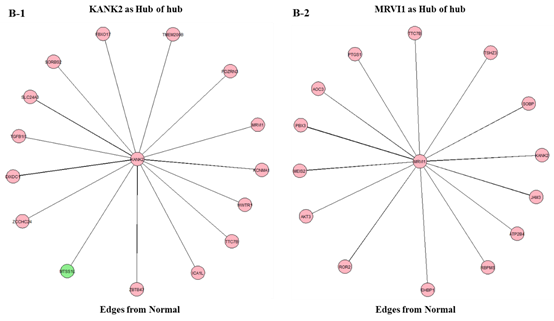


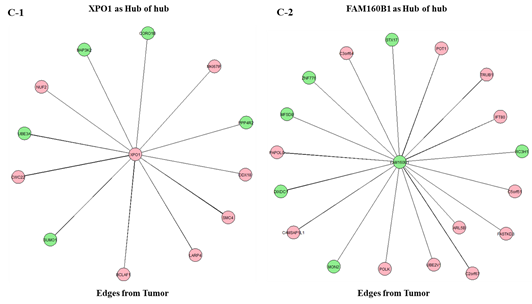


**Supplementary Figure 8. Representative six hubs of hub genes with its edge genes from only hub genes calculated using the degree centrality analysis of the normal and tumor groups.** Two hub of hub genes in both groups (A), only in the normal group (B), and only in the tumor group (C). Green fill indicates downregulated genes in the DEG analysis, red fill indicates upregulated genes in the DEG analysis, and red font indicates common genes in both groups. Edge width: coefficient power


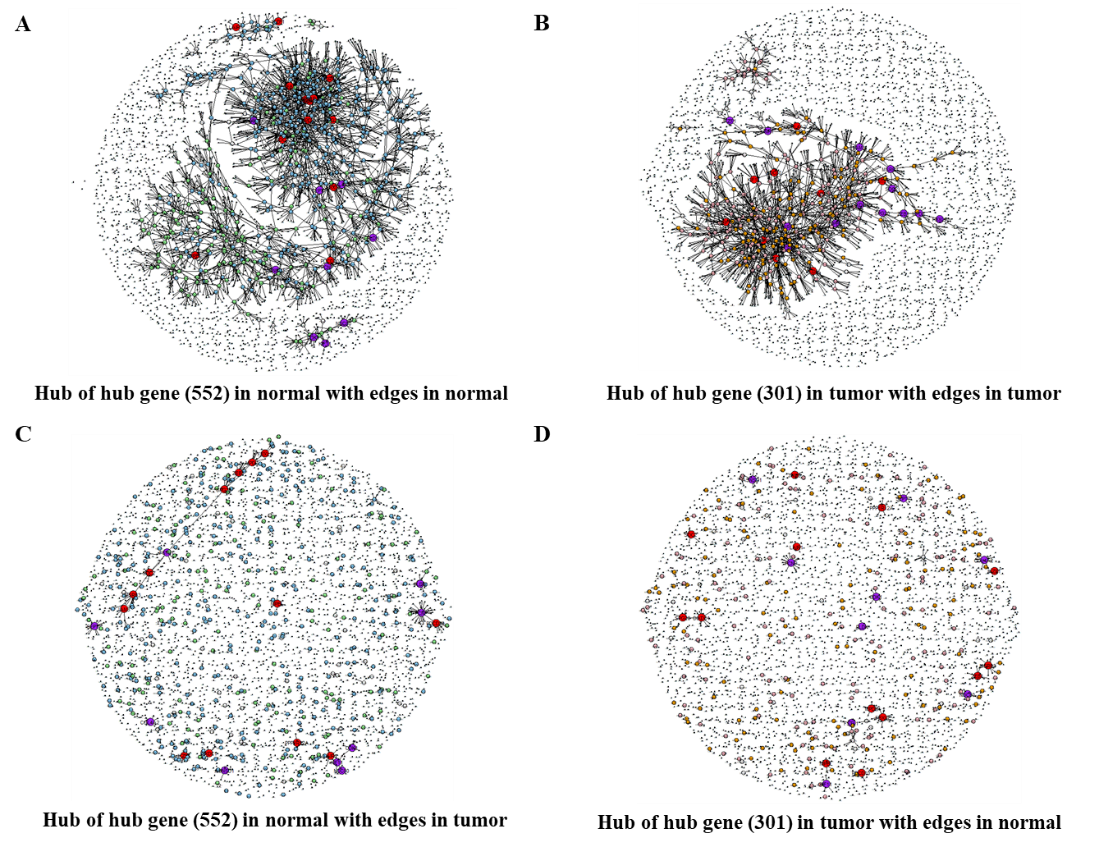


**Supplementary Figure 9. Network analysis of the hub of hub genes with edges from the hub genes and edges from the original edges of the hub genes in each group.** Hub of hub genes (552) of the normal group applied into the normal group including hub genes as well as original edges (Supplementary table 3A) of hub genes. B. Hub of hub genes (301) in the tumor group applied into the tumor group including hub genes as well as original edges (Supplementary table 3B) from hub genes. C. Hub of hub genes (552) in the normal group applied to the tumor group including hub genes as well as original edges (Supplementary table 3B) of hub genes. D. Hub of hub genes (301) in the tumor group applied to the normal group including hub genes as well as original edges (Supplementary table 3A) of hub genes. The list of gene set in the network included all hub genes and their edges from both the normal and tumor groups, but the position of each gene is not same in A, B, C, and D. The largest ball indicates the common hub of hub genes, the medium-sized ball indicates hub genes, and the smallest ball indicates edges genes of hub genes. Lines indicate the connection between genes. Purple indicates common hubs of hub genes that are upregulated in the tumor group (downregulated in the normal group). Red indicates the common hubs of hub genes that are downregulated in the tumor group (downregulated in the normal group). Green indicates hub genes in the normal group that are downregulated in the normal group (upregulated in the tumor group). Sky blue indicates hub genes in the normal group that are upregulated in the normal group (downregulated in the tumor group). Pink indicates hub genes in the tumor group that are upregulated in the tumor group (downregulated in the normal group). Orange indicates hub genes in the tumor group that are downregulated in the tumor group (upregulated in the normal group). White indicates edge genes.

Supplementary Figure 10


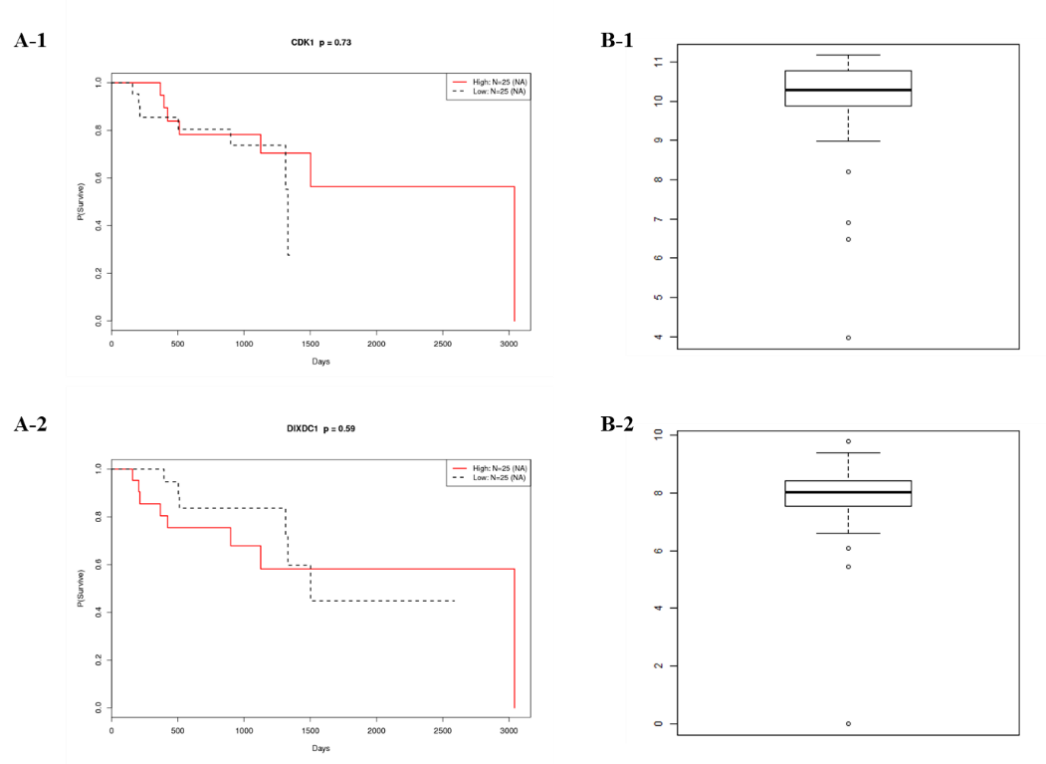

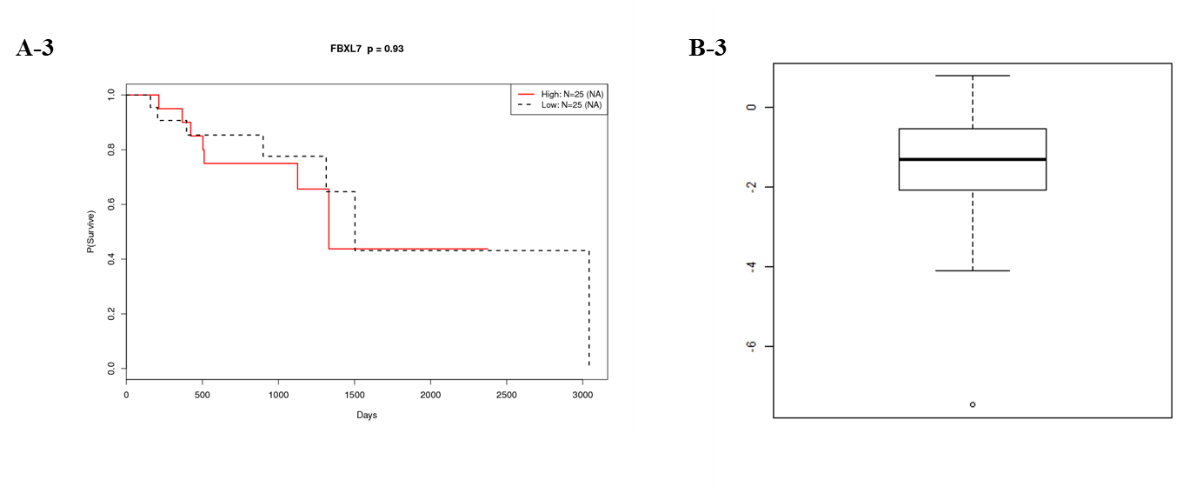

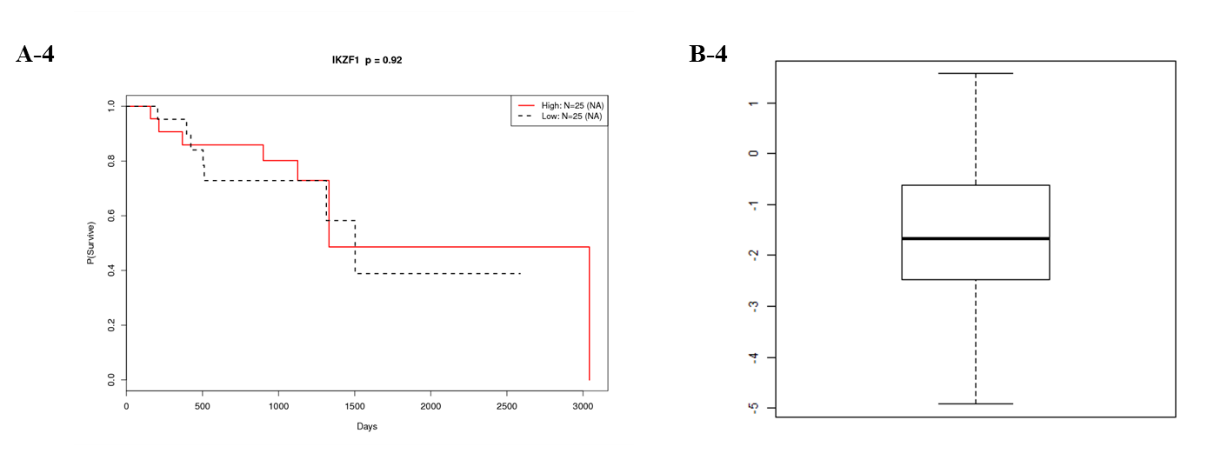


Supplementary Figure 10


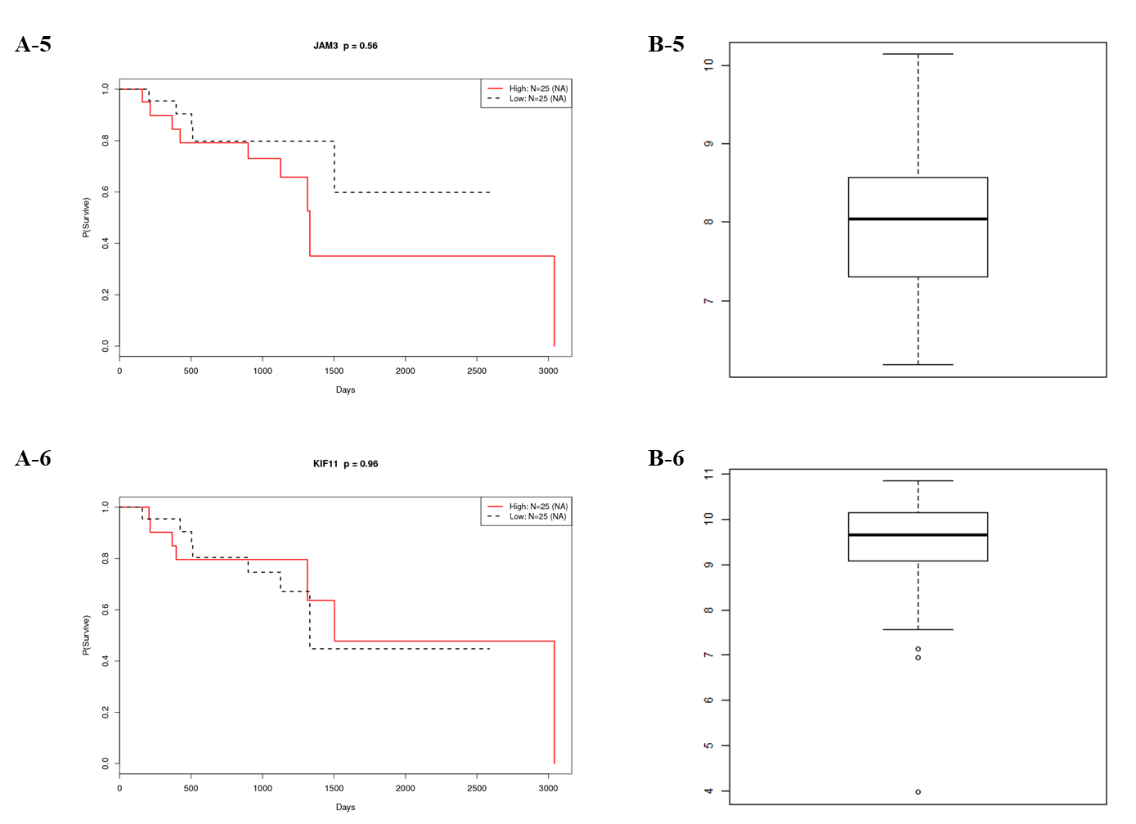

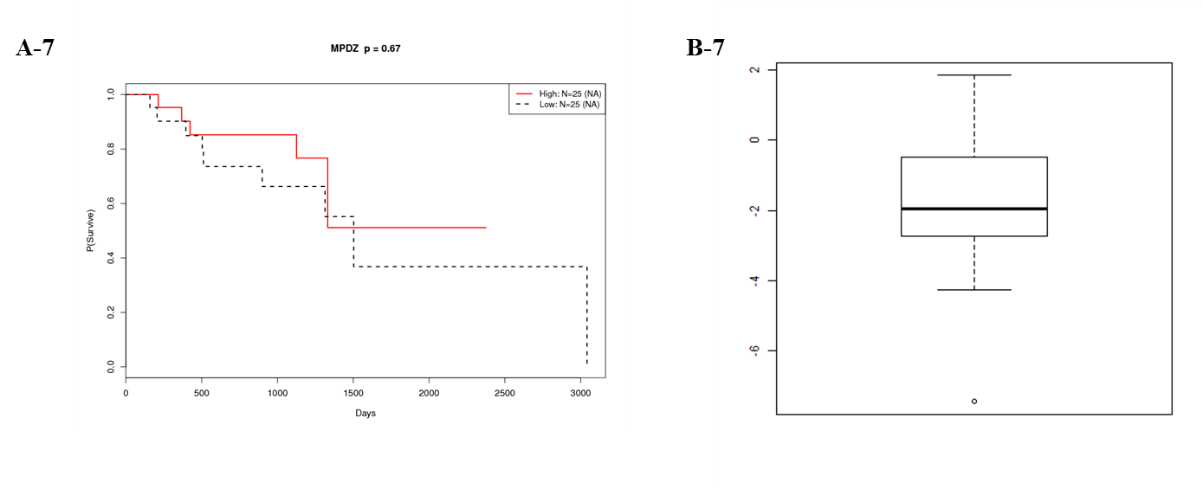

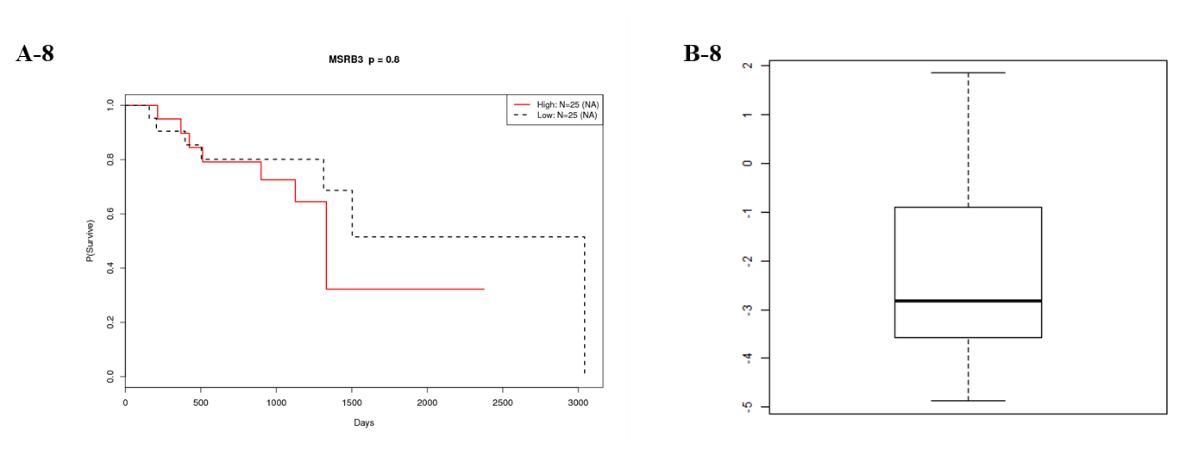


Supplementary Figure 10


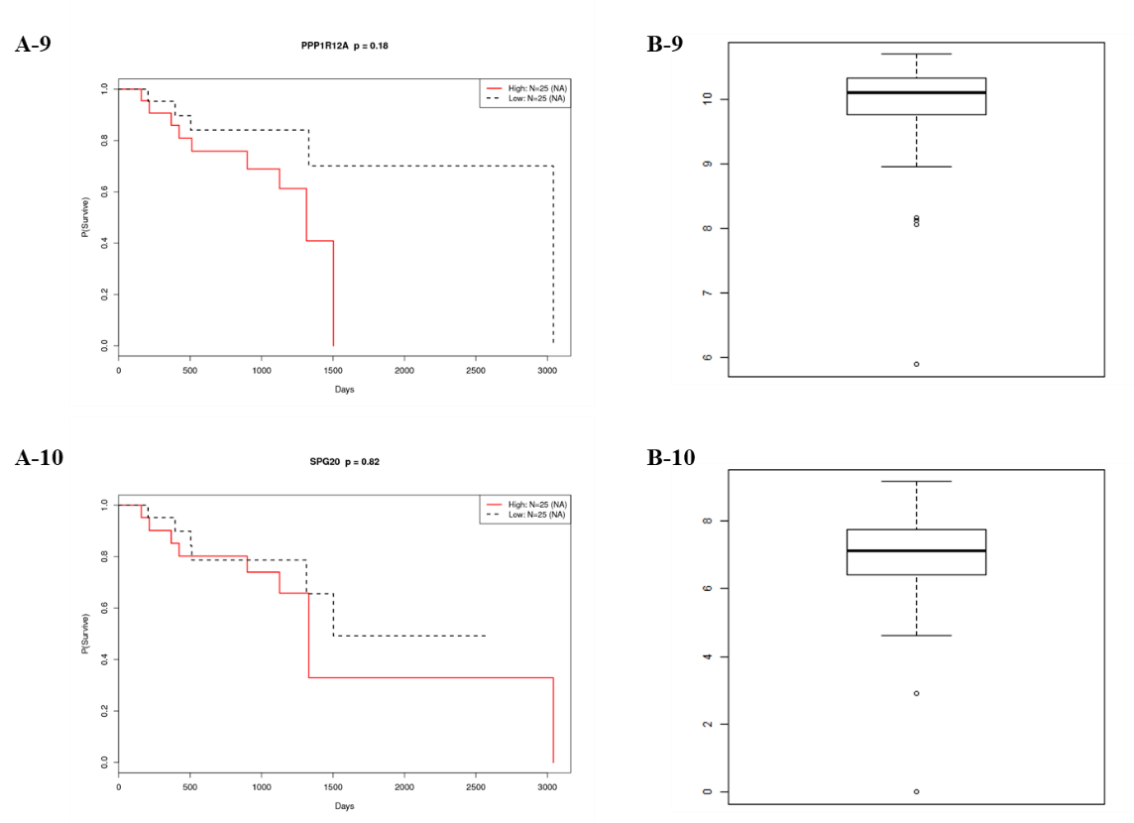

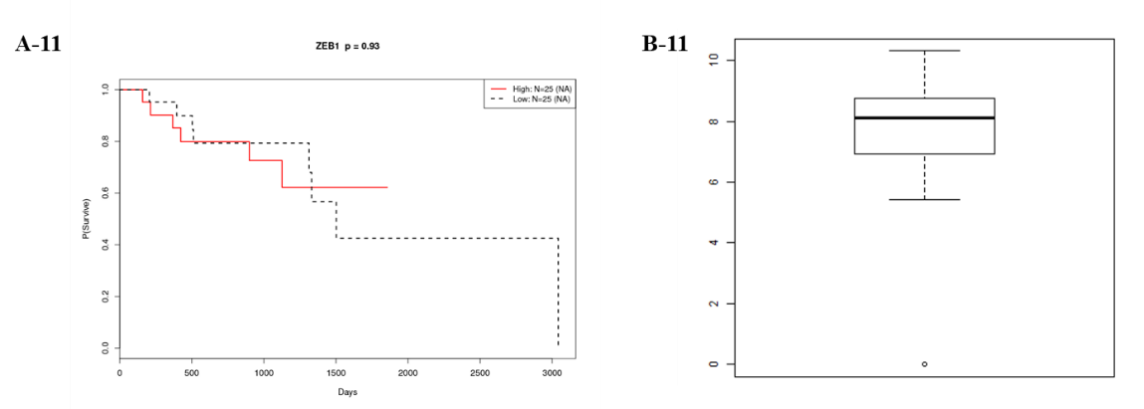


**Supplementary Figure 10. Kaplan–Meier survival curve of the selected hub genes.** A. Survival curve for each gene, B. Boxplot with DEGs for each gene.

**3. Supplementary Table List**

**Supplementary Table 1. 9,427 genes selected (p-value < 0.001, p-value < adj_p-value) from DEG analysis of the tumor and their paired normal group with colorectal cancer collected from TCGA.** Bold genes indicate the gene with the highest p-value drawn in the heat map. * indicates adj_p-value = i/m×FDR Level, (i: rank, m: the number of total variable, FDR Level: 0.001).

**Supplementary Table 2A. Degree of centrality analysis of the hub genes (4,627) with their edges from the normal group.**

**Supplementary Table 2B. Degree of centrality analysis of the hub genes (2,680) with their edges from the tumor group.**

**Supplementary Table 3A. Degree centrality analysis of the selected hub (602) genes with their edges in the normal group after cutting off based on the mean weight (≥0.0679) and average degree (≥ 3.5) for further analysis.** Green filling indicates 25 common hub genes between the normal (Supplementary Table 3A) and tumor (Supplementary Table 3B) groups. Orange filling indicates common edges in common hub between the normal (Supplementary Table 3A) and tumor (Supplementary Table 3B) groups. Hub genes in bold (181) indicate common the hub genes between Supplementary Table 3A and 3A-1. Blue genes in bold indicate common edges of common hub genes between Supplementary Table 3A and 3A-1.

**Supplementary Table 3A-1. Degree of centrality analysis of the 602 hub genes for the normal group (Supplementary Table 3A) in the tumor group (Supplementary Table 2B).** Hub genes in bold (181) indicate common hub genes between Supplementary Table 3A and 3A-1. Blue genes in bold indicate common edges of common hub genes between Supplementary Table 3A and 3A-1**.**

**Supplementary Table 3B. Degree centrality analysis of the selected hub (328) genes with their edges in the tumor group after cutting off based on weight (≥0.0603) and average degree (≥ 3.4) for further analysis.** Green filling indicates 25 common hub genes between the normal group (Supplementary Table 3B) and the tumor group (Supplementary Table 3A). Orange filling indicates common edges in the common hub between the normal (Supplementary Table 3B) and tumor (Supplementary Table 3A) groups. Hub genes in bold (127) indicate common hub genes between Supplementary Table 3B and 3B-1. Blue genes in bold indicate common edges of common hub genes between Supplementary Table 3B and 3B-1.

**Supplementary Table 3B-1. Degree of centrality analysis of the 328 hub genes for the tumor group (Supplementary Table 3B) in the normal group (Supplementary Table 2A).** Hub genes in bold (127) indicate common hub genes between Supplementary Table 3B and 3B-1. Bolded blue genes indicate common edges of common hub genes between Supplementary Table 3B and 3B-1.

**Supplementary Table 4. Summary of common and different hub genes in the tumor and paired normal groups.**

**Supplementary Table 5A. Degree of centrality analysis of the selected 552 hub of hub genes with their edge genes from only the 602 hub genes by discounting their edge genes in the normal group.** Green filling indicates 22 common hub genes between the normal (Supplementary Table 5A) and tumor (Supplementary Table 5B) groups. Orange filling indicates common edges in the common hub between the normal (Supplementary Table 5A) and tumor (Supplementary Table 5B) groups.

**Supplementary Table 5B. Degree of centrality analysis of the selected 301 hub of hub genes with their edge genes from only the 321 hub genes by discounting their edge genes in the tumor group.** Green filling indicates 22 common hub genes between the normal (Supplementary Table 5A) and tumor (Supplementary Table 5B) groups. Orange filling indicates common edges in the common hub between the normal (Supplementary Table 5A) and tumor (Supplementary Table 5B) groups.

**Supplementary Table 6. DEG of hub genes in the normal and tumor groups.** Yellow filling indicates 503 significant genes obtained using the FDR level (+: p ≤ 0.001 & p ≤ p_adj_). Red indicates 25 common hub genes in the normal and tumor groups. 0 indicates no hub in each group. * indicates adj p-value. adj p-value=i/m×FDR Level, (i: rank, m: the number of total variable, FDR Level: 0.001).

**Supplementary Table 7. Description and references of selected hub genes by comparison DEG sets and hub genes from each group.**

**Supplementary Table 8. Survival analysis of the selected hub genes.**

**Supplementary Table 9. List of hub genes investigated (602 of hub genes from normal and 328 of hub genes from tumor) by STRING analysis (Confidence score ≥ 0.95).** Yellow filling indicates DEG significance (p ≤ 0.001 & p ≤ p_adj_).

**Supplementary Table 10. Enriched GO terms obtained upon searching 602 hub genes from the normal and 328 hub genes from the tumor groups.** * [Number of hub genes involved in this pathway/number of total hub genes from the normal or tumor groups] × 100.

**Supplementary Table 11. Enriched pathways based on KEGG pathway analysis of the 602 hub genes from the normal and 328 hub genes from the tumor.** * [Number of hub genes involved in this pathway/number of total hub genes from normal or tumor] × 100. * [Number of hub genes involved in this pathway/number of total hub genes from normal or tumor] × 100.
